# Trivalent Rare‐Earth Metal Amide Complexes as Catalysts for the Hydrosilylation of Benzophenone Derivatives with HN(SiHMe_2_)_2_ by Amine‐Exchange Reaction

**DOI:** 10.1002/chem.202002011

**Published:** 2020-10-01

**Authors:** Koichi Shinohara, Hayato Tsurugi, Reiner Anwander, Kazushi Mashima

**Affiliations:** ^1^ Department of Chemistry Graduate School of Engineering Science Osaka University Toyonaka Osaka 560-8531 Japan; ^2^ Institut für Anorganische Chemie Universität Tübingen Auf der Morgenstelle 18 72076 Tübingen Germany

**Keywords:** amine exchange, hydrosilylation, rare-earth metals, Si-H beta-agostic interactions

## Abstract

The rare‐earth metal complexes Ln(**L^1^**)[N(SiHMe_2_)_2_](thf) (Ln=La, Ce, Y; **L^1^**=*N*,*N′′*‐bis(pentafluorophenyl)diethylenetriamine dianion) were synthesized by treating Ln[N(SiHMe_2_)_2_]_3_(thf)_2_ with **L^1^**H_2_. The lanthanum and cerium derivatives are active catalysts for the hydrosilylation of benzophenone derivatives with HN(SiHMe_2_)_2_. An amine‐exchange reaction was revealed as a key step of the catalytic cycle, in which Ln−Si−H β‐agostic interactions are proposed to promote insertion of the carbonyl moiety into the Si−H bond.

## Graphical abstract

**Lanthanum and cerium complexes** with doubly deprotonated *N*,*N*′′‐bis(pentafluorophenyl)diethylenetriamines as ligands catalyze the hydrosilylation of benzophenone derivatives with HN(SiHMe_2_)_2_ to afford the corresponding products, HN[Si(OCHAr_2_)Me_2_]_2_. Activation of the Si−H bonds of [N(SiHMe_2_)_2_] ligand via Ln−Si−H β‐agostic interactions was crucial for accelerating carbonyl insertion to form Si−O bonds. A kinetic study revealed amine exchange to be the turnover limiting step.

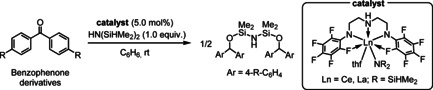

## Introduction

The reduction of carbonyl compounds is one of the most important and straightforward synthetic transformations in organic chemistry for producing the corresponding alcohol.^[1]^ Metal hydrides of transition metals and some main‐group elements such as (*i*Bu)_2_AlH (DIBAL) have been used as versatile and stoichiometric reducing reagents;^[2]^ however, the strong reducing ability of these metal hydrides often causes low substrate selectivity. Catalytic hydrosilylation is an attractive alternative that has been widely studied by using noble metal catalysts (e.g., Rh, Ir, and Pt).^[3]^ A recent trend has been directed to the use of base metal catalysts, including first‐row transition metal complexes (e.g., Fe, Co, and Ni),^[4]^ but no rare‐earth metal complexes have yet been applied as catalysts for the hydrosilylation of carbonyl groups, presumably due to their high oxophilicity to form stable Ln−O bonds.^[5]^ In fact, although lanthanide hydrides are known to be applicable for catalytic hydrosilylation of alkenes^[6]^ and alkynes,^[7]^ there are limited examples of the transformation of lanthanide alkoxides to lanthanide hydrides in the presence of hydrosilanes with elimination of silyl ethers.[^6f^, ^6g^]

Aiming at the hydrosilylation of carbonyl compounds by using any lanthanide catalyst, we were especially interested in amine‐exchange reactions of lanthanide amide complexes with primary or secondary amines by a σ‐bond metathesis pathway. In fact, σ‐bond metathesis reactions of lanthanide amide complexes with amines are well known to proceed catalytically in hydroamination^[8]^ and other reactions.^[9]^ Thus, 1,1,3,3‐tetramethyldisilazane, HN(SiHMe_2_)_2_, was selected as a silane reagent; the cerium complex Ce(**L^1^**)[N(SiHMe_2_)_2_](thf) (**L^1^**=*N*,*N′′*‐bis(pentafluorophenyl)diethylenetriamine dianion) was found to perform best in the hydrosilylation of benzophenone and its derivatives. The catalytic cycle was revealed to feature an amine‐exchange reaction as a key step. In addition, the Si−H moiety of the N(SiHMe_2_)_2_ ligand seems to be activated by the Lewis acidic lanthanide metal center and accelerate carbonyl insertion into the Si−H bonds to form Si−O bonds.

## Results and Discussion

The search for an efficient lanthanide catalyst system for the hydrosilylation of benzophenone (**2 a**) with 1 equiv of HN(SiHMe_2_)_2_ was started by using an in situ mixture of *N*,*N′′*‐bis(pentafluorophenyl)diethylenetriamine (**L^1^**H_2_) (5.0 mol %) with an equimolar amount of the rare‐earth metal amido compounds Ln[N(SiHMe_2_)_2_]_3_(thf)_2_ (**1 a**: Ln=La; **1 b**: Ln=Ce; **1 c**: Ln=Nd; **1 d**: Ln=Gd; **1 e**: Ln=Lu; **1 f**: Ln=Y), in benzene at ambient temperature for 3 h, and the results are summarized in Table 1. The lanthanum complex **1 a** catalyzed the hydrosilylation of **2 a** to give HN[Si(OCHPh_2_)Me_2_]_2_ in 90 % yield (entry 1), and the cerium complex **1 b** gave even a slightly higher yield of the product (94 % yield, entry 2). The ion size of the metal sensitively affected the catalytic performance: complex **1 c** with the moderately‐sized neodymium center exhibited markedly lower catalytic activity (22 % yield, entry 3). Complexes of the even smaller‐sized gadolinium complex **1 d**, lutetium **1 e**, and yttrium **1 f** afforded HN[Si(OCHPh_2_)Me_2_]_2_ in 9, 10, and 10 % yields, respectively (entries 4–6). The significant effect of the metal size in the catalytic activity was due to the suppression of the amine exchange step during the catalytic cycle (vide infra). Using the best catalytic system of **L^1^**H_2_ and the cerium triamide complex **1 b**, we examined other silanes, such as PhSiH_3_, Ph_2_SiH_2_, (EtO)_3_SiH, O(SiHMe_2_)_2_, and polymethylhydrosiloxane (PMHS), as hydrosilylation reagents instead of HN(SiHMe_2_)_2_, but all exhibited low catalytic activities (entries 7–11), which was ascribed to the importance of Ln−Si−H agostic interaction for the carbonyl insertion into the Si−H bond (vide infra). Therefore, we selected the cerium triamide **1 b** as the best catalyst precursor and HN(SiHMe_2_)_2_ as the best silane reagent.

**Table 1:** Screening of the catalyst system.^[a]^

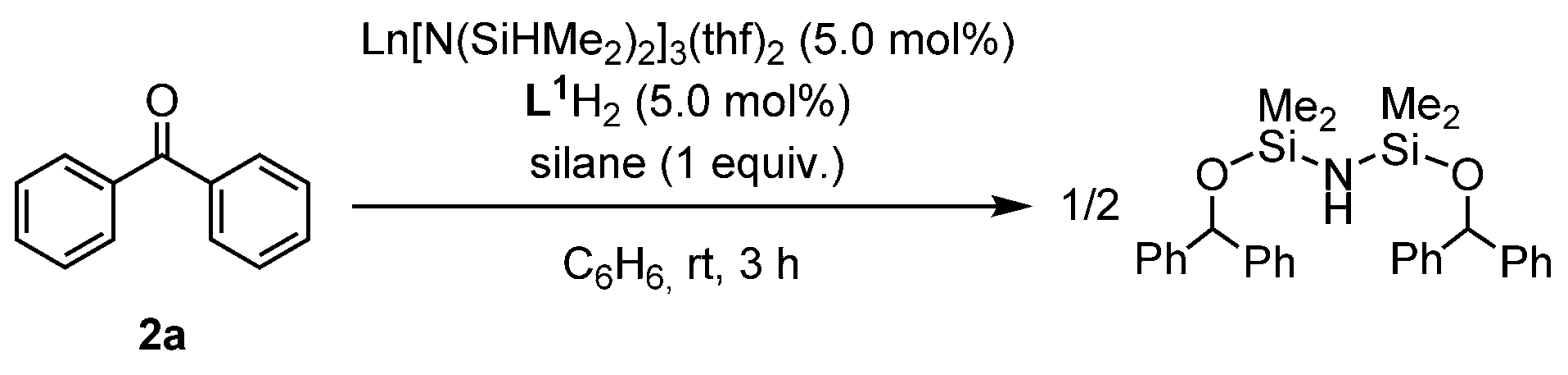			
	Ln	Silane	Yield^[b]^ [%]
1	La (**1 a**)	HN(SiHMe_2_)_2_	90
2	Ce (**1 b**)	HN(SiHMe_2_)_2_	94
3	Nd (**1 c**)	HN(SiHMe_2_)_2_	22
4	Gd (**1 d**)	HN(SiHMe_2_)_2_	9
5	Lu (**1 e**)	HN(SiHMe_2_)_2_	10
6	Y (**1 f**)	HN(SiHMe_2_)_2_	10
7^[c]^	Ce (**1 b**)	PhSiH_3_	15
8^[c]^	Ce (**1 b**)	Ph_2_SiH_2_	25
9^[c]^	Ce (**1 b**)	(EtO)_3_SiH	12
10	Ce (**1 b**)	O(SiHMe_2_)_2_	11
11^[c]^	Ce (**1 b**)	PMHS	14

[a] Reaction conditions: benzophenone (0.100 mmol), Ln[N(SiHMe_2_)_2_]_3_(thf)_2_ (0.005 mmol), **L^1^**H_2_ (0.005 mmol), C_6_H_6_ (0.5 mL), HN(SiHMe_2_)_2_ (0.100 mmol). [b] ^1^H NMR yield using 1,3,5‐trimethoxybenzene as an internal standard. [c] Product was a mixture of HN[Si(OCHPh_2_)Me_2_]_2_ and corresponding silylated alcohols derived from the used silanes.

Next, we checked some amine proligands, including tridentate, bidentate, and monodentate nitrogen proligands, under the same catalytic conditions by using **1 b** and HN(SiHMe_2_)_2_ (Table 2). We started by using diethylenetriamine derivatives **L^1^**H_2_‐**L^4^**H_2_. In sharp contrast to the high yield (94 %) for **L^1^**H_2_, the use of proligand **L^2^**H_2_, an *N′*‐methylated derivative of **L^1^**H_2_, afforded HN[Si(OCHPh_2_)Me_2_]_2_ in 14 % yield (entry 2). When *N*,*N′′*‐bis(2,6‐dimethylphenyl)diethylenetriamine (**L^3^**H_2_) and its *N′*‐methylated derivative (**L^4^**H_2_) were used as supporting ligands, HN[Si(OCHPh_2_)Me_2_]_2_ was obtained in moderate yields (49 and 48 %, respectively; entries 3 and 4). Bidentate *N*,*N′*‐substituted ethylenediamine derivatives, such as **L^5^**H_2_ with pentafluorophenyl groups, **L^6^**H_2_ with 2,6‐dimethylphenyl groups, **L^7^**H_2_ with 2,6‐diisopropylphenyl groups, **L^8^**H_2_ with 2‐methylphenyl groups, and **L^9^**H_2_ with cyclohexyl groups, exhibited low to moderate catalytic activities (entries 5–9). Monodentate nitrogen proligands (5.0 mol %), pentafluoro‐*N*‐ethylaniline (**L^10^**H), dibenzylamine (**L^11^**H), and dicyclohexylamine (**L^12^**H), afforded HN[Si(OCHPh_2_)Me_2_]_2_ in 21, 54, and 51 % yields, respectively (entries 10–12). When the loadings of monodentate ligands, **L^11^**H_2_ and **L^12^**H_2_, were increased up to 10 mol %, the yield of HN[Si(OCHPh_2_)Me_2_]_2_ was slightly increased (entries 10–12, parentheses). Without any nitrogen proligand, Ce[N(SiHMe_2_)_2_]_3_(thf)_2_ (**1 b**) solely produced HN[Si(OCHPh_2_)Me_2_]_2_ in 58 % yield (entry 13).^[10]^ Accordingly, we selected a mixture of **L^1^**H_2_ and **1 b** as the best catalyst combination.

**Table 2:** Screening of nitrogen proligands for the cerium‐catalyzed hydrosilylation of benzophenone.^[a]^

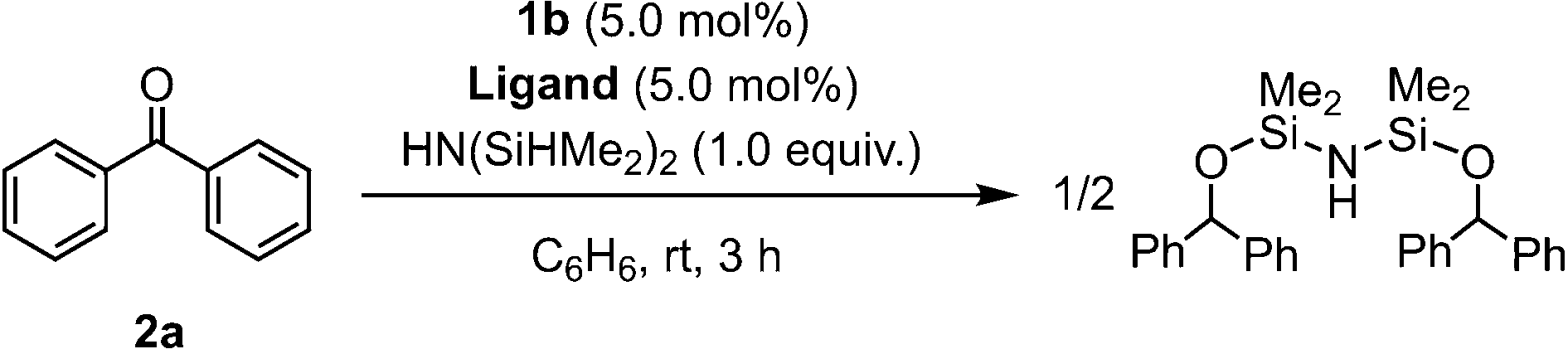					
	Proligand	Yield^[b]^ [%]		Proligand	Yield^[b]^ [%]
1	**L^1^**H_2_	94	8	**L^8^**H_2_	43
2	**L^2^**H_2_	14	9	**L^9^**H_2_	26
3	**L^3^**H_2_	49	10	**L^10^**H_2_	21 (26)^[c]^
4	**L^4^**H_2_	48	11	**L^11^**H_2_	54 (56)^[c]^
5	**L^5^**H_2_	17	12	**L^12^**H_2_	51 (57)^[c]^
6	**L^6^**H_2_	34	13	–	58
7	**L^7^**H_2_	54			
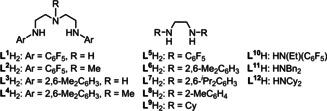					

[a] Reaction conditions: benzophenone (0.100 mmol), **1 b** (0.005 mmol), proligand (0.005 mmol), C_6_H_6_ (0.5 mL), HN(SiHMe_2_)_2_ (0.100 mmol). [b] ^1^H NMR yield using 1,3,5‐trimethoxybenzene as an internal standard. [c] 10 mol % of proligands.

Treatment of **L^1^**H_2_ with **1 b** in toluene at ambient temperature for 1 h gave cerium complex **4 b** in 87 % yield [Eq. 1]. The complex **4 b** was characterized by spectral data and X‐ray analysis. Due to the paramagnetic nature of the cerium(III) center, the ^1^H NMR spectrum of **4 b** in C_6_D_6_ displayed two broad singlets centered at *δ*=11.95 and −8.63, assignable to ethylene protons of the ligand and a broad singlet at *δ*=−19.03 for the amine proton of the ligand; in addition, the Si*H* and Si*Me_2_* signals of the N(SiHMe_2_)_2_ moiety were observed as two broad singlets at *δ*=−54.20 and 2.60, respectively. The large high‐field shift of the Si*H* hydrogen atom is particularly prominent and indicative of a pronounced Ce−Si−H β‐agostic interaction. For comparison, the chemical shifts of and Si−H moieties in Ce[N(SiHMe_2_)_2_]_3_(thf)_2_ (**1 b**), Ce[N(SiHMe_2_)_2_]_4_Li(thf)^[11]^ and Cp*_2_Ce[N(SiHMe_2_)_2_]^[12]^ were observed at −5.62, −17.70, and −28.63 ppm, respectively. One set of signals for two C_6_F_5_ groups for **4 b** was observed at *δ*=−165.6, −178.5, and −182.7 for *meta*, *ortho*, and *para*‐fluorine atoms in the ^19^F NMR spectrum, respectively. The signal for *ortho*‐fluorine atoms was broadened, which was ascribed to the interaction of the *ortho*‐fluorine atom to the paramagnetic cerium(III) center. The C−F−Ce^III^ interaction in solution was further investigated by the variable temperature ^19^F NMR measurement in [D_8_]toluene: de‐coalescence of a signal assignable to the *meta*‐fluorine atoms was observed upon cooling the measurement temperature to −70 °C. Based on the ^19^F NMR spectra of **4 b**, the energy barrier for the rotation of the C_6_F_5_ around the *N*−C_ipso_ bonds was estimated to be 4.6 kcal mol^−1^.
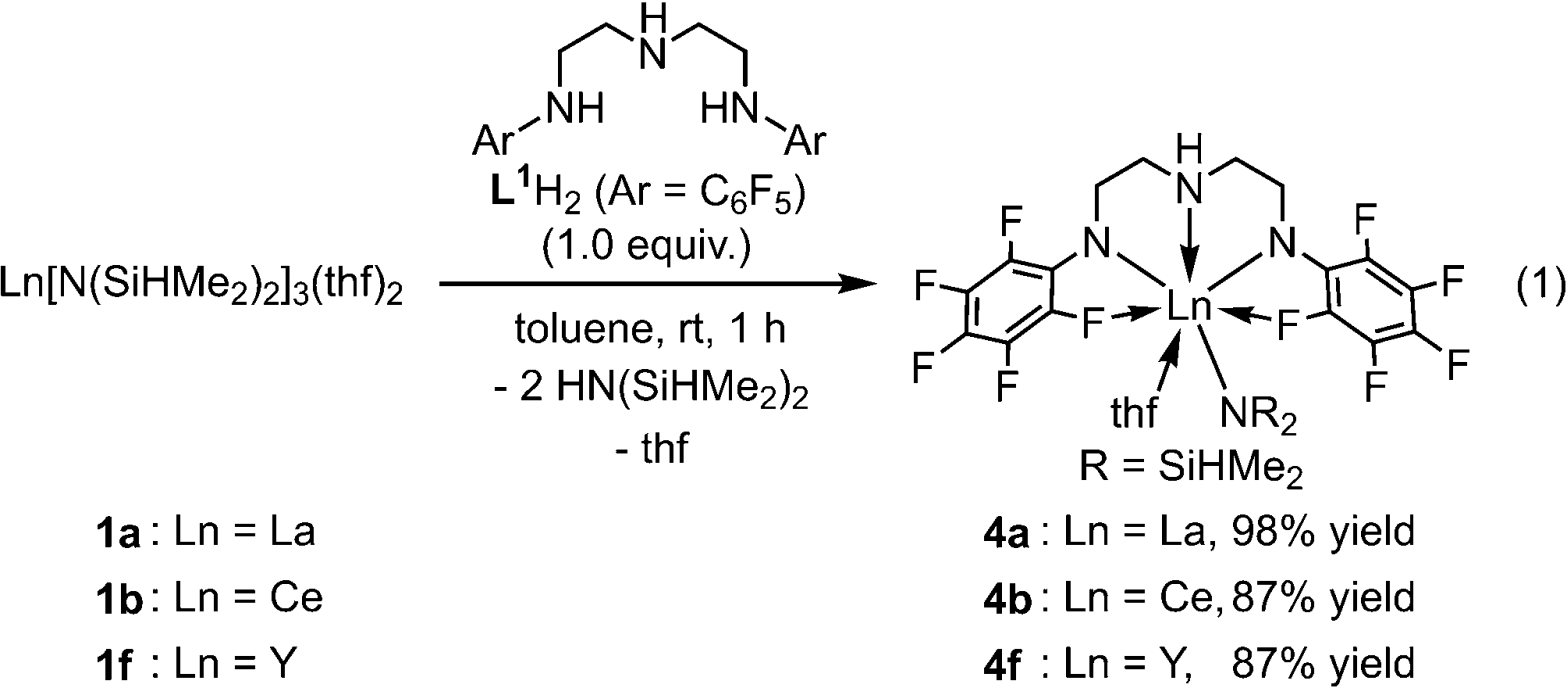



We applied isolated complex **4 b** as a catalyst for the hydrosilylation of **2 a** using HN(SiHMe_2_)_2_ at ambient temperature in C_6_H_6_ for 5 h to give diphenylmethanol (**3 a**) after acidic work‐up in 97 % yield. Accordingly, we applied **4 b** as the catalyst for further hydrosilylation reactions of *para*‐substituted benzophenones (**2 b**–**k**) using HN(SiHMe_2_)_2_ as a silane reagent (Table 3). Benzophenone derivatives such as **2 b** with *para*‐fluoro, **2 c** with *para*‐chloro, **2 d** with *para*‐bromo, and **2 e** with *para*‐iodide were applicable for the reaction, giving the corresponding alcohols **3 b** (98 %), **3 c** (98 %), **3 d** (93 %), and **3 e** (91 %) in high yields without any loss of the C−X bonds (entries 2–5). *Para*‐trifluoromethyl‐substituted benzophenone derivative **2 f** afforded the corresponding alcohol **3 f** in 60 % yield (entry 6). Benzophenone derivatives having an electron‐donating substituent at the *para*‐position needed longer reaction time for giving the high yield of corresponding hydrosilylated products because of their low reactivity for the Si‐H insertion step: **2 g** with a methyl group, **2 h** with a *tert*‐butyl group, and **2 i** with a methoxy group, afforded the corresponding products **3 g**, **3 h**, and **3 i** in 94, 97, and 79 % yields, respectively, after 20 h (entries 7–9). *para*‐Amino functionalities for **2 j** and **2 k** suppressed the catalytic hydrosilylation even heated at 60 °C due to the electron‐rich character of the carbonyl moiety (entries 10 and 11). In sharp contrast to benzophenone derivatives, reaction of HN(SiHMe_2_)_2_ with benzaldehyde selectively afforded a Tishchenko reaction product, benzyl benzoate, in a catalytic manner (53 % yield after 24 h), having similar reactivity to the previously reported homoleptic lanthanide silylamide complexes.^[13]^ When acetophenone was used as a substrate, **4 b** was rapidly decomposed, presumably due to enolate formation by deprotonation at the α‐position of the carbonyl group, whereas the reaction with cyclopropyl phenyl ketone afforded cyclopropyl‐(phenyl)methanol in 67 % yield after the acidic work‐up.

**Table 3:** Catalytic hydrosilylation of *para*‐substituted benzophenones.^[a]^

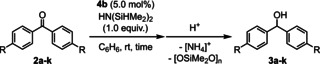			
	R	*t* [h]	Yield^[b]^ [%]
1	H (**2 a**)	5	97
2	F (**2 b**)	5	98
3	Cl (**2 c**)	5	98
4	Br (**2 d**)	5	93
5	I (**2 e**)	5	91
6	CF_3_ (**2 f**)	5	60
7	Me (**2 g**)	20	94
8	^*t*^Bu (**2 h**)	20	97
9	OMe (**2 i**)	20	79
10^[c]^	NH_2_ (**2 j**)	20	n.d.
11^[c]^	NMe_2_ (**2 k**)	20	n.d.

[a] Reaction conditions: substrate (0.100 mmol), **4 b** (0.005 mmol), C_6_H_6_ (0.5 mL), HN(SiHMe_2_)_2_ (0.100 mmol). [b] ^1^H NMR yield of corresponding alcohol after acidic work‐up (by HCl aq.) using 1,3,5‐trimethoxybenzene or hexamethylbenzene as an internal standard. [c] 60 °C; n.d.: not determined.

**Figure 1:**
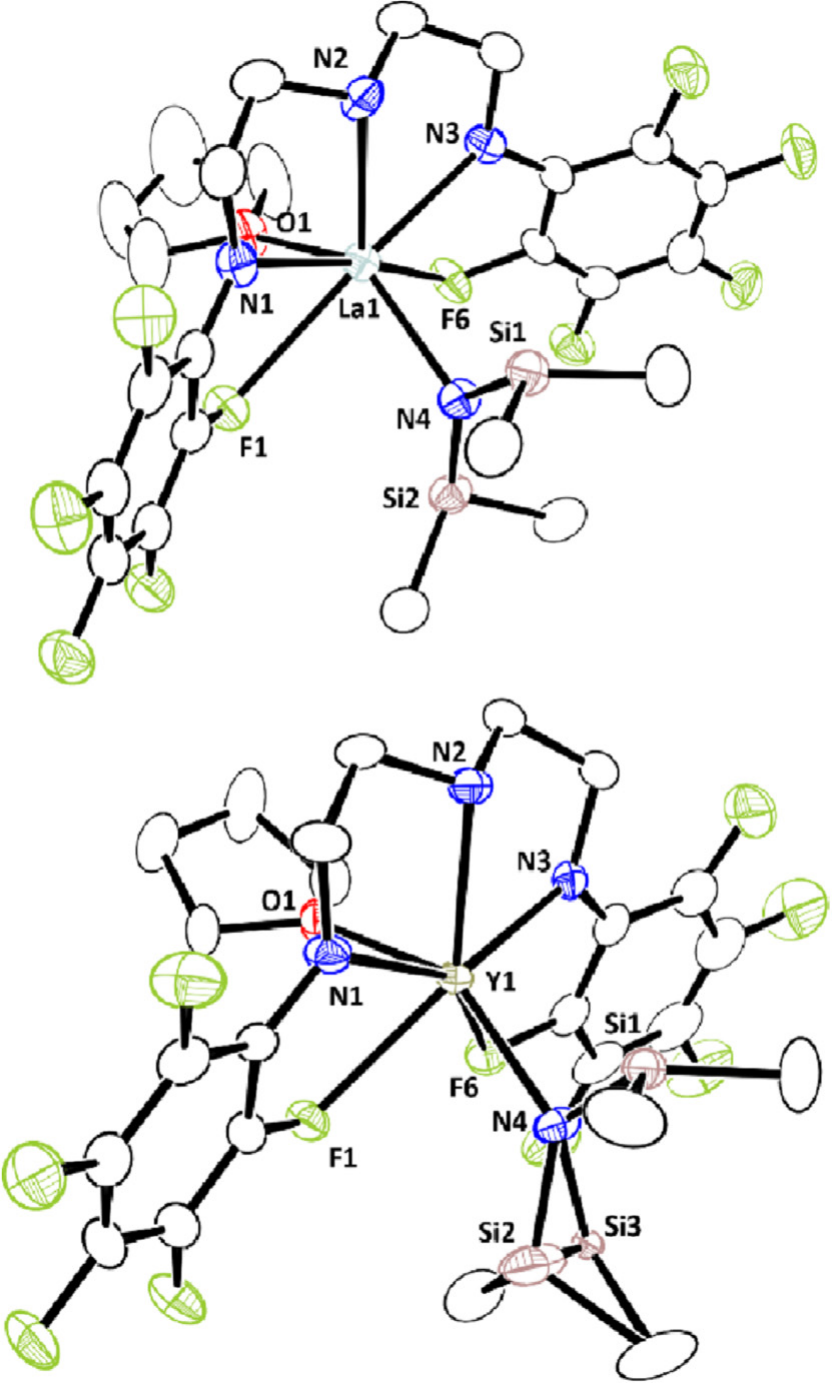
ORTEP drawings of the crystal structures of **4 a** (top) and **4 f** (bottom) with ellipsoids shown at 50 % probability. All hydrogen atoms and solvent molecules are omitted for clarity.

To gain insight into the reaction mechanism by means of NMR spectroscopy, due to the paramagnetic nature of **4 b**, we prepared the diamagnetic complexes of lanthanum and yttrium bearing **L^1^** according to the reaction as shown in eq 1: treatment of La[N(SiHMe_2_)_2_]_3_(thf)_2_ (**1 a**) and Y[N(SiHMe_2_)_2_]_3_(thf)_2_ (**1 f**) with **L^1^**H_2_ in toluene at ambient temperature for 1 h afforded **4 a** (98 %) and **4 f** (87 %) [Eq. (1)]. Both of the complexes were fully characterized by NMR and IR spectroscopies, and X‐ray diffraction analyses. The ^1^H NMR spectrum of **4 a** in C_6_D_6_ at ambient temperature exhibited only one signal for two Si−H bonds at *δ*_Si−H_=4.75, which is comparable to that detected at *δ*_Si−H_=4.70 for two Si−H bonds in **4 f**. The Si*H* signals in **4 a** and **4 f** did not de‐coalesce, even at 193 K. Furthermore, the smaller Si‐H coupling constant in **4 a** (^1^ *J*_SiH_=153 Hz) than that in **4 f** (^1^ *J*_SiH_=164 Hz) indicated that the Si−H bonds of **4 a** are weaker than those of **4 f** in solution. The IR spectrum of **4 a** showed a broad absorption for the agostic Si−H stretching vibration at 2002 cm^−1^, while **4 f** showed two well‐separated Si−H stretching vibrations at 2051 and 2107 cm^−1^, assignable to an agostic Si−H bond and a non‐agostic Si−H bond.^[14]^ Figure 1 shows the crystal structures of **4 a** and **4 f**, and selected interatomic distances and angles between the metal center and bis(dimethylsilyl)amido moiety are summarized in Table 4. Both the lanthanum atom in **4 a** and the yttrium atom in **4 f** adopt a 7‐coordinated distorted pentagonal bipyramidal geometry with a nitrogen atom of a N(SiHMe_2_)_2_ ligand and an oxygen atom of THF at the apical positions, while three nitrogen atoms of the ligand **L^1^** and two fluorine atoms at the *ortho*‐position of two *N*‐C_6_F_5_ groups coordinate at the equatorial sites. Noteworthy is the structural difference between **4 a** and **4 f**: lanthanum complex **4 a** has a double β‐Si‐H agostic interaction featured by a large Si1‐N4‐Si2 angle of 137.7(4)°, whereas yttrium complex **4 f** has a single β‐Si‐H agostic interaction with a narrower Si1‐N4‐Si2 angle of 123.3°.^[14]^ The geometric parameters for **4 b** are essentially the same as those for **4 a** due to the similar ionic radius of lanthanum and cerium. The structural differences in **4 a**, **4 b**, and **4 f** having different ionic radii of the central metal affect the reactivity of their Si−H bonds toward the benzophenone insertion and amine exchange reaction (vide infra).

**Table 4:** Selected structural parameters for lanthanide silylamide complexes **4 a** and **4 f** bearing ligand **L^1^**.

Distances [Å]			Angles [°]		
	**4 a**	**4 f**		**4 a**	**4 f**
M–Si1	3.250(2)	3.078(2)	M‐N4‐Si1	105.6(3)	101.2(2)
M–Si2	3.519(3)	3.652(av.)	M‐N4‐Si2	119.7(3)	132.3(av.)
M–N4	2.360(6)	2.266(5)	Si1‐N4‐Si2	137.7(4)	123.3(av.)

The lanthanum complex **4 a** exhibited almost the same catalytic activity (**3 a** produced in 99 % yield) as **4 b**, while the catalytic activity of **4 f** was very low (**3 a** produced in 9 % yield). Because we obtained both highly (La) and marginally (Y) active diamagnetic complexes, we examined these complexes **4 a** and **4 f** in terms of their stoichiometric reactions with benzophenone. We first carried out the reactions of **4 a** and **4 f** with 2 equivalents of **2 a** to give the corresponding benzophenone‐inserted complexes **5 aa** (96 %) and **5 fa** (77 %) [Eq. 2]. Both diamagnetic complexes **5 aa** and **5 fa** were characterized by ^1^H, ^19^F, and ^13^C NMR analysis.
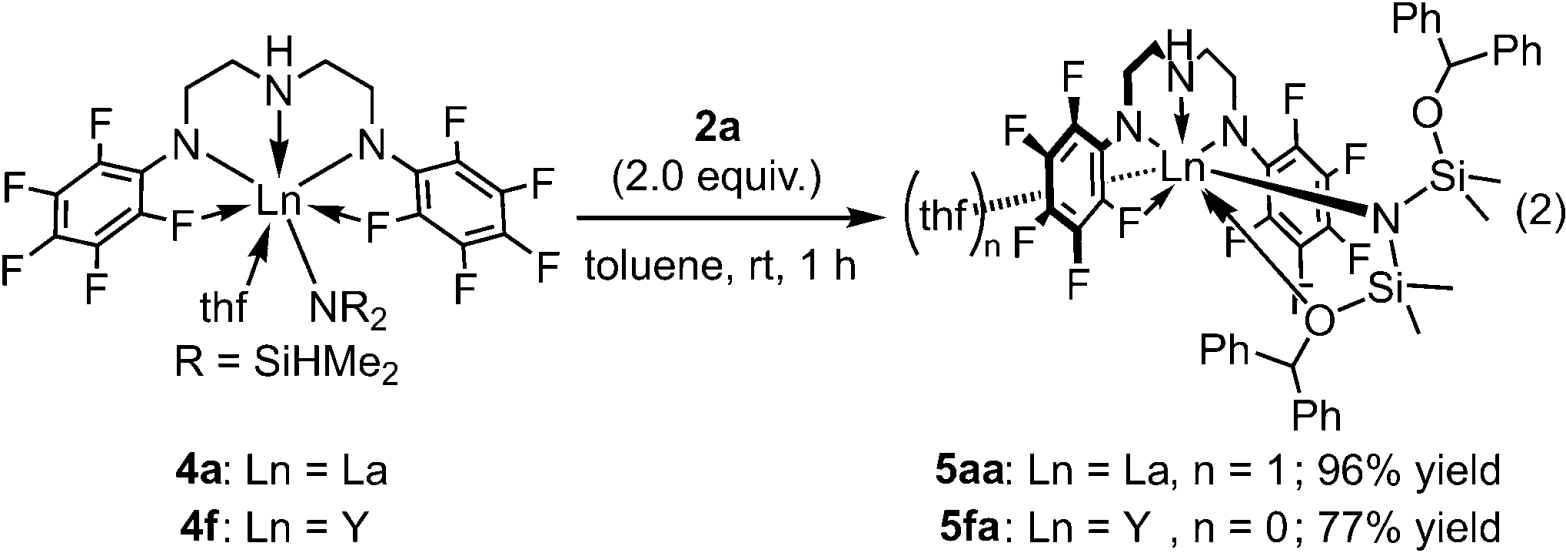



**Figure 2:**
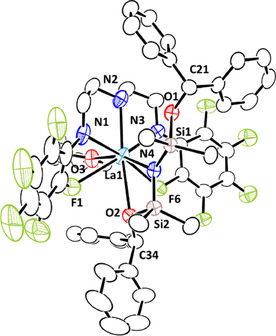
ORTEP drawing of the crystal structure of **5 aa** with ellipsoids shown at 50 % probability. All hydrogen atoms and solvent molecules are omitted for clarity. Selected interatomic distances [Å]: La1–N1, 2.545(5); La1–N2, 2.600(5); La1–N3, 2.520(5); La1–N4, 2.405(4); La1–F1, 2.698(3); La1–F6, 2.792(4); La1–O1, 3.549(3); La1–O2, 2.850(4); C21–O1, 1.441(6); C34–O2, 1.440(6). Selected interatomic angles [°]: La1‐N4‐Si1, 108.9(2); La1‐N4‐Si2, 123.9(2).

Figure 2 shows the crystal structure of **5 aa**, and the structure of the cerium analogue **5 ba** is included in the Supporting Information. The lanthanum atom adopts an 8‐coordinated geometry with three nitrogen atoms of **L^1^**, two fluorine atoms at the *ortho*‐positions of two *N*‐C_6_F_5_ groups, and one oxygen atom of the two siloxy moieties in the equatorial plane of the distorted hexagonal bipyramid, and a nitrogen atom of N[Si(OCHPh_2_)Me_2_]_2_ and an oxygen atom of THF located at the apical sites. The distances of La−N1 (2.545(5) Å), La−N3 (2.520(5) Å), and La−N4 (2.405(4) Å) are slightly longer than the corresponding distances in complex **4 a** due to the steric hindrance caused by N[Si(OCHPh_2_)Me_2_]_2_. One of two diphenylmethoxy groups coordinates to the lanthanum center, which increases the steric crowding around the lanthanum atom. The distances for O1−C21 (1.441(6) Å) and O2−C34 (1.440(6) Å) are typical C−O single bonds, consistent with the double‐hydrosilylation of benzophenone by two Si−H bonds in **4 a**. With respect to carbonyl‐inserted silylamide complexes, formaldehyde‐ and acetone‐inserted zirconium cationic complexes, [Cp_2_ZrN(SiMe_2_OR)_2_]^+^ (R=Me and *i*Pr),^[15]^ and an acetophenone‐inserted yttrium complex, Y[N(SiMe_2_OCHMePh)Dipp][N(SiHMe_2_)Dipp]_2_,^[16]^ were reported.

**Scheme 1:**
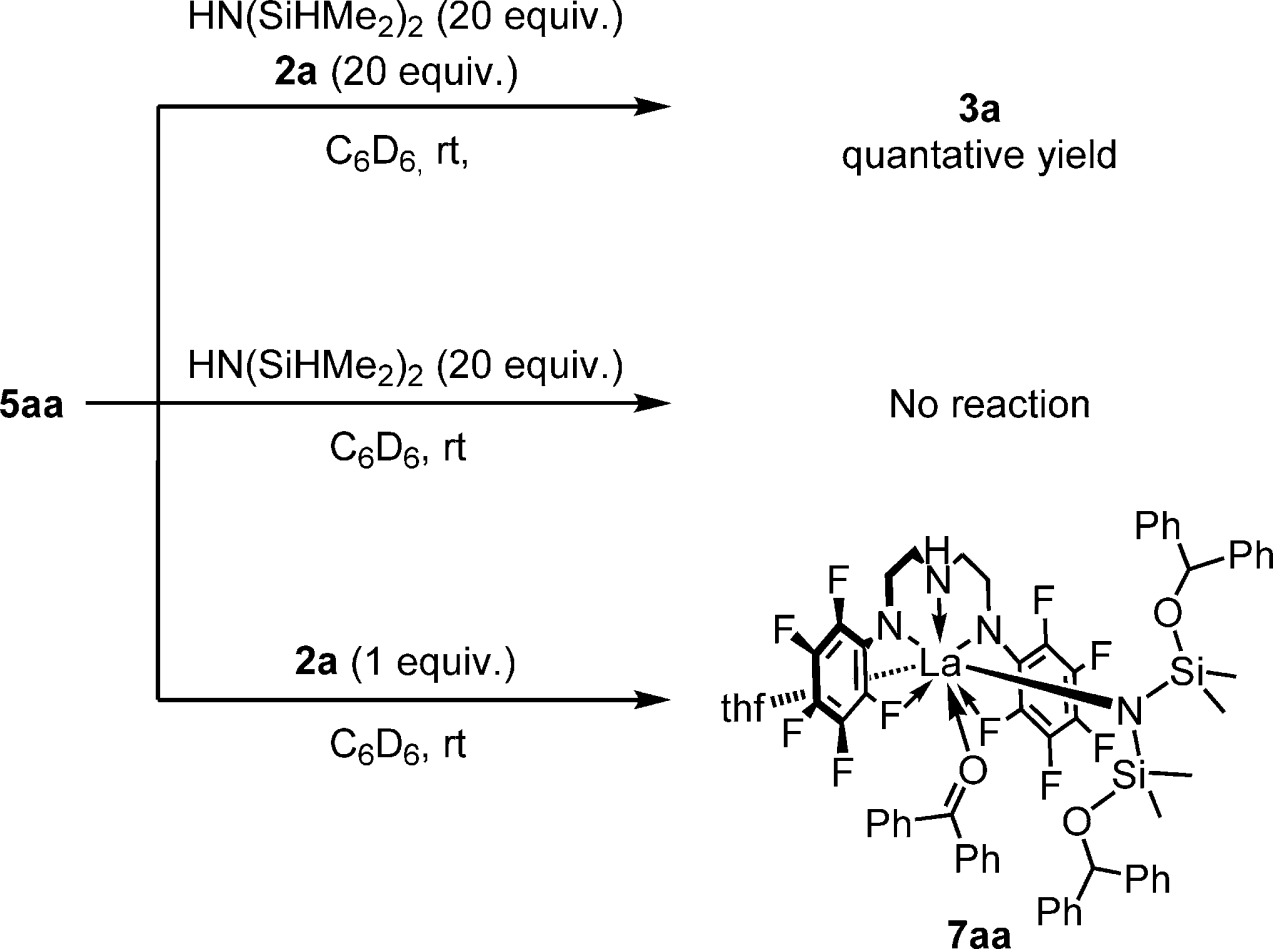
Ligand‐exchange reaction of **5 aa** with HN(SiHMe_2_)_2_ in the presence (top) and in the absence (middle) of **2 a**. The formation of benzophenone adduct **7 aa** (bottom).

To investigate the details of a carbonyl insertion reaction for **4 a** and **4 f**, we used di‐*tert*‐butyl benzophenone **2 h** instead of **2 a** because the insertion rate of **2 a** was too fast to trace the reaction profiles. The reaction of **4 a** with two equivalents of **2 h** proceeded at 265 K to give **5 ah** in 78 % yield together with a monohydrosilylated complex **6 ah** in 20 % yield after 70 min, while the reaction of **4 f** was much slower, giving **5 fh** in 43 % yield and **6 fh** in 27 % yield with remaining **4 f** (30 %). Such distinct reactivity of **4 a** and **4 f** might be due to a metal size effect as reflected by a double agostic interaction in **4 a** and a single agostic interaction in **4 f** (vide supra). We further conducted an amine exchange reaction of **5 aa** with HN(SiHMe_2_)_2_ in the presence of **2 a**, which released the double‐hydrosilylated product, HN[Si(OCHPh_2_)Me_2_]_2_, in quantitative yield (Scheme 1, above). Crucially, the absence of **2 a** did not lead to an amine exchange reaction between **5 aa** and HN(SiHMe_2_)_2_ (Scheme 1, middle), indicating that coordination of the benzophenone substrate is indispensable for the replacement of the amido ligand N[Si(OCHPh_2_)Me_2_]_2_ in **5 aa** with HN(SiHMe_2_)_2_ in this catalytic hydrosilylation reaction. In fact, addition of 1 equivalent of **2 a** to **5 aa** in C_6_D_6_ quantitatively afforded benzophenone adduct **7 aa** (Scheme 1, below).^[17]^ Coordination of benzophenone derivatives to a lanthanide center was reported for Ln[N(SiMe_3_)_2_]_3_ (Ln=Ce, Pr).^[17a]^ In contrast, yttrium complex **5 fa** did not engage in an amine exchange reaction despite the presence or absence of benzophenone because of the lack of enough coordination sites for the smaller yttrium center than lanthanum, in accordance with the different catalytic activity observed for lanthanum complex **4 a** and yttrium complex **4 f**. Complexes **5 aa** and **7 aa** were stable in solution, suggesting no involvement of amine elimination by the central N−H moiety of the tridentate ligand.

We conducted a kinetic study for the hydrosilylation of **2 a** by HN(SiHMe_2_)_2_ under optimized reaction conditions, in which the isolated lanthanum complex **4 a** was used as a catalyst, by means of variable time normalization analysis.^[18]^ The obtained rate law is provided in Equation 3. The reaction obeyed a first‐order with respect to both of the concentration of **4 a** and the concentration of HN(SiHMe_2_)_2_, suggesting that the amine exchange reaction of **5 aa** with HN(SiHMe_2_)_2_ is the rate‐determining step in this catalytic reaction. On the other hand, the reaction was inverse second‐order by the concentration of **2 a**, indicating that coordination of **2 a** to the metal center significantly impaired the insertion of the carbonyl moiety into the Si−H bond (vide infra).

(3) $$\mathrm{Reaction}\;\mathrm{rate}\;\propto \;\frac{\left[4a\right][{\mathrm{HN}(\mathrm{SiH}{\mathrm{Me}}_{2})}_{2}]}{{\left[2a\right]}^{2}}\;$$

**Scheme 2:**
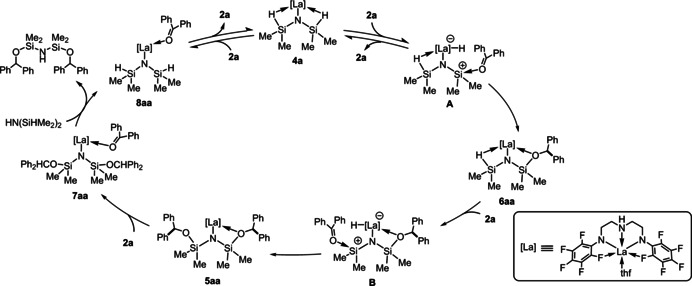
A plausible mechanism for the lanthanum‐catalyzed hydrosilylation of **2 a** with HN(SiHMe_2_)_2_.

**Figure 3:**
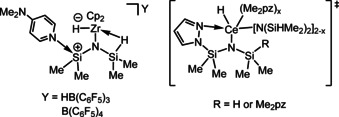
Interaction between a Lewis base and electrophilic silicon atoms of disilylamide ligands in hydride complexes.

As shown in Scheme 2, on the basis of the above spectroscopic analysis as well as stoichiometric reactions together with a kinetic study, we propose a plausible catalytic cycle for the hydrosilylation of **2 a** with HN(SiHMe_2_)_2_ using **4 a**. First, **4 a** reacts with 1 equivalent of **2 a**, giving monohydrosilylated complex **6 aa** via interaction of the carbonyl moiety with the cationic silicon center of **A**:[^4p^, ^19^] interaction of Lewis bases with the silicon atom of the M−Si−H β‐agostic species due to the increased Lewis acidity of the silicon atom was reported for a cationic zirconium bis(dimethylsilyl)amide complex with 4‐(dimethylamino)pyridine by Sadow et al. (Figure 3, left)^[15a]^ and a cerium complex bearing a dimethylpyrazolyl‐substituted silylamido ligand (Figure 3, right).^[20a]^ Further, another equivalent of **2 a** spontaneously reacts with an Si−H bond in **6 aa** through intermediate **B** to form double‐hydrosilylated complex **5 aa**. Coordination of **2 a** to the metal center causes a dissociation of the diphenylmethoxy moiety in **5 aa**, thereby forming **7 aa**, which undergoes an amine exchange reaction with HN(SiHMe_2_)_2_ to afford the double‐hydrosilylated product HN[Si(OCHPh_2_)Me_2_]_2_ along with **8 aa**. Liberation of **2 a** from **8 aa** regenerates the catalytically active complex **4 a**, though this pathway is unfavorable when the concentration of **2 a** is high, based on the kinetic study.^[19]^ In fact, a **2 k**‐coordinated adduct **8 ak**, analogue of **8 aa**, was detected via the ^1^H and ^19^F NMR spectra for the reaction mixture of **4 a** and *para*‐dimethylamino benzophenone (**2 k**), though no insertion of **2 k** into the Si−H bond was observed. Disappearance of the β‐Si‐H agostic interaction in **8 ak**, confirmed by the significant downfield shift of the Si−H proton signal (*δ=*5.25) and the increased ^1^ *J*_SiH_ coupling (160 Hz), indicates that the carbonyl moiety of benzophenone derivatives inserts into the Si−H bond activated by the metal center.^[14]^

## Conclusions

Rare‐earth metal silylamide complexes Ln(**L^1^**)[N(SiHMe_2_)_2_](thf) (Ln=La, Ce) bearing an *N*,*N′′*‐bis(pentafluorophenyl)diethylenetriamine dianionic ligand **L^1^** promote the hydrosilylation of benzophenone derivatives with HN(SiHMe_2_)_2_. Control experiments and kinetic studies revealed a plausible reaction mechanism, in which amine exchange of the benzophenone‐inserted amido moiety with bis(dimethylsilyl)amine plays a key role in obtaining the desired doubly hydrosilylated product. We are currently investigating the scope of such rare‐earth metal‐catalyzed reductions of unsaturated compounds involving these amine‐exchange pathways.

## Conflict of interest

The authors declare no conflict of interest.

## Supporting information

As a service to our authors and readers, this journal provides supporting information supplied by the authors. Such materials are peer reviewed and may be re‐organized for online delivery, but are not copy‐edited or typeset. Technical support issues arising from supporting information (other than missing files) should be addressed to the authors.

Supplementary
