# Supplementary material for: Trivalent Rare‐Earth Metal Amide Complexes as Catalysts for the Hydrosilylation of Benzophenone Derivatives with HN(SiHMe2)2 by Amine‐Exchange Reaction
Source: Chemistry. 2020 Oct 1;26(62):14130–6. doi: 10.1002/chem.202002011 (PMC7745047; doi:10.1002/chem.202002011)
Supplement: Supplementary file 1 — Supplementary [file CHEM-26-14130-s001.pdf]

Chemistry–A European Journal

Supporting Information

Trivalent Rare-Earth Metal Amide Complexes as Catalysts for the Hydrosilylation of Benzophenone Derivatives with $HN(SiHMe_2)_2$ by Amine-Exchange Reaction

Koichi Shinohara,[a] Hayato Tsurugi,*[a] Reiner Anwander,*[b] and Kazushi Mashima*[a]

Supporting Information

for

Trivalent Rare-earth Metal Amide Complexes as Catalysts for Hydrosilylation of Benzophenone Derivatives with $HN(SiHMe_2)_2$ *via* Amine Exchange Reaction

Koichi Shinohara,† Hayato Tsurugi,*1,† Reiner Anwander,*2,‡ and Kazushi Mashima*3,†

*1tsurugi@chem.es.osaka-u.ac.jp, *2reiner.anwander@uni-tuebingen.de,
*3mashima@chem.es.osaka-u.ac.jp

† Department of Chemistry, Graduate School of Engineering Science, Osaka University, Toyonaka, Osaka 560-8531, Japan.

‡ Institut für Anorganische Chemie, Universität Tübingen, Auf der Morgenstelle 18, 72076, Germany

Contents

I. General Procedure S2
II. Synthesis of *N,N'*-Bis(pentafluorophenyl)ethylendiamine L^5H_2 .... S3
III. Procedure for Catalytic Hydrosilylation of Benzophenone Derivatives S3
IV. Hydrosilylation of 2a Catalyzed by $Ln[N(SiHMe_2)_2]_3(thf)_2$ S5
V. Preparation of Lanthanide Dimethylsilylamide Complexes S6
VI. VT-^{19}F NMR Spectra of 4b in toluene-*d*8 .... S19
VII. Hydrosilylation of 2a Catalyzed by 4a, 4b, and 4f S20
VIII. Time Course Profiles for Formation of Benzophenone-inserted Complexes 5ah and 5fh S21
IX. Reaction of 4a and 4f with 1 equiv of 2a S23
X. Reaction of Double Hydrosilylated Complexes 5aa and 5fa with 1 equiv of 2a S25
XI. Kinetic Study S27
XII. The Formation of 2k-Coordinated Adduct 8ak S30
XIII. Spectral Data for Diarymethanol Derivatives S31
XIV. ^{1}H NMR Spectra of Catalytic Reaction Mixtures S32
XV. Crystal Data and Data Collection Parameters S37
XVI. Molecular Structure of 4b and 5ba S39

I. General Procedure

All manipulations involving air- and moisture-sensitive compounds were carried out under an argon atmosphere by using standard vacuum line and Schlenk tube techniques or Ar-filled glovebox. Anhydrous toluene and hexane were purchased (KANTO CHEMICAL CO., INC., Purity(GC): min. 99.5 %) and purified by passing through Grubbs column (Glass Counter Solvent Dispending System, Nikko Hansen & Co, Ltd.).[S1] Toluene-d_8, C_6D_6, and benzene were purchased and purified by distillation over CaH_2. $Ln[N(SiHMe)_2]_3(thf)_2$,[S2] $\mathbf{L^1}H_2$,[S3] $\mathbf{L^2}H_2$,[S4] $\mathbf{L^3}H_2$,[S5] $\mathbf{L^4}H_2$,[S6] $\mathbf{L^6}H_2$,[S7] $\mathbf{L^7}H_2$,[S8] $\mathbf{L^8}H_2$,[S8] and $\mathbf{L^9}H_2$ [S8] were prepared according to the literature. $\mathbf{L^{10}}H_2$ [S9] was synthesized according to modified literature procedures. Benzophenone derivatives such as **2e**,[S10] **2g** [S11] and **2h**[S12] were synthesized according to the literature. All other reagents were purchased at the highest commercial quality and used without further purification. ^{1}H NMR (400 MHz), ^{13}C{^{1}H} NMR (100 MHz), ^{19}F NMR (376 MHz), and ^{29}Si NMR (79 MHz) spectra were measured on Bruker Avance III-400 spectrometers in 5 mm NMR tubes. All ^{1}H NMR chemical shifts were reported in ppm relative to the residual solvent protons in chloroform-d_1 at δ 7.26, benzene-d_6 at δ 7.16, and toluene-d_8 at δ 2.08. All ^{13}C{^{1}H} NMR chemical shifts were reported in ppm relative to carbon resonance of the solvent itself in benzene-d_6 at δ 128.06. High-resolution mass spectrometry (HRMS) was performed on a JEOL JMS-700 (EI, FAB plus) and a Brucker Daltonics MicroTOF (ESI plus). All X-ray crystallographic studies were performed on Rigaku XtaLAB P200 system with graphite-monochromated Mo Kα radiation (λ = 0.71075). PLATON/SQUEEZE[S13] program was used for the refinement of **4f**. Alert B was still remained due to the disorder of $N(SiHMe_2)_2$ moiety and solvent molecules as well as residual electron density for the heavy lanthanide atom for **5aa**. All melting point were recorded on BUCHI melting point M-565. Elemental analyses were recorded by using Perkin-Elmer 2400 at the Faculty of Engineering Science, Osaka University.

II. Synthesis of *N,N'*-Bis(pentafluorophenyl)ethylendiamine L^5H_2

Hexafluorobenzene (5.58 g, 30.0 mmol) was added to a CH_3CN solution (10 mL) of ethane-1,2-diamine (0.67 mL, 10.0 mmol) and K_2CO_3 (3.10 g, 22.4 mmol). The mixture was heated to reflux for 11 days. The reaction mixture was cooled to ambient temperature, and water (100 mL) was added. Organic component was extracted with chloroform (50 mL x 3). The pale-yellow extract was dried over $MgSO_4$ and concentrated to give yellow oil. Subsequent purification filtered through a pad of silica gel using EtOAc:Hexane (v:v = 2:8) as eluent gave 1.51 g of $\mathbf{L^5}H_2$ as a colorless solid (38% yield). **^{1}H NMR** (400 MHz, $CDCl_3$): δ 3.53 (s, 4H, CH_2), 3.77 (brs, 2H, N*H*). **^{19}F NMR** (376 MHz, $CDCl_3$): δ -159.2 (td, 4F, *ortho*), -164.0 (t, 4F, *meta*), -170.3 (tt, 2F, *para*). **^{13}C NMR**, (100 MHz, C_6D_6): δ 46.7, 139.7, 137.2, 134.3 (d, $^1J_{CF}$ = 244 Hz), 123.5. **HRMS (FAB) (m/z)**: $[M]^+$ calcd. for $C_{14}H_7F_{10}N_2$, 392.0371; found, 392.0371.

III. Procedure for Catalytic Hydrosilylation of Benzophenone Derivatives

III-1. Hydrosilylation of **2a** Catalyzed by an *in-situ* Mixture of Pro-ligand with $Ln[N(SiHMe_2)_2]_3(thf)_2$

In an Ar-filled glove box, a collection vial was charged with $Ln[N(SiHMe_2)_2]_3(thf)_2$ (0.005 mmol), pro-ligand (0.005 mmol), and C_6H_6 (0.25 mL). After a few minutes, a solution of benzophenone (0.100 mmol) in benzene (0.25 mL) and silane (0.100 mmol) were added. The reaction mixture was stirred at ambient temperature in a glovebox. After stirring the reaction mixture for 3 h, the collection vial was quickly removed from the glovebox, and then ether was added for quenching the reaction mixture. Internal standard was added, and all volatiles were removed under reduced pressure. The residue was dissolved in $CDCl_3$, and the yield of the hydrosilylated products, were determined by ^{1}H NMR measurement. When using $PhSiH_3$, Ph_2SiH_2, $(EtO)_3SiH$, and PMHS as a silane reagent, product was a mixture of $HN[Si(OCHPh_2)Me_2]_2$ and corresponding silylated alcohols, such as $PhSiH_2$-$OCHPh_2$, Ph_2SiH-$OCHPh_2$, $(EtO)_3Si$-$OCHPh_2$, and $[MeSi(OCHPh_2)O]_n$, respectively.

III-2. Hydrosilylation of Benzophenone Derivatives Catalyzed by **4b**

In an Ar-filled glove box, a collection vial was charged with **4b** (0.005 mmol) and C_6H_6 (0.25 mL). A solution of benzophenone derivatives (0.100 mmol) in benzene (0.25 mL) and $HN(SiHMe_2)_2$ (17.3 µL, 0.100 mmol) were added. The reaction mixture was stirred at ambient temperature in a glovebox. After stirring the reaction mixture for 5 or 20 h, the collection vial was quickly removed from the glovebox, and then ether (1.5 mL) and aqueous HCl (3 M, 2 mL) were added with vigorous stirring. Organic compounds were extracted with ether (2 mL x 3), and internal standard was added. All volatiles were removed under reduced pressure. The residue was dissolved in $CDCl_3$, and the yield of the corresponding alcohol was determined by ^{1}H NMR measurement. (The signal intensity of HOC*H*Ar_2 was calculated with respect to the internal standard.)

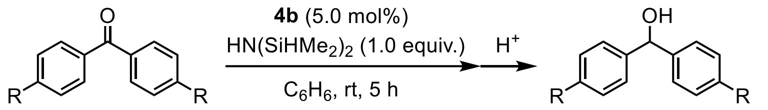

2a-k **3a-k**

entry	R	Yield [%]	TOF [h^{-1}]
1	H (**2a**)	97	3.9
2	F (**2b**)	98	3.9
3	Cl (**2c**)	98	3.9
4	Br (**2d**)	93	3.7
5	I (**2e**)	91	3.6
6	CF_3 (**2f**)	60	2.4
7	Me (**2g**)	39 (94)[a]	1.6 (0.9)[a]
8	^{t}Bu (**2h**)	58 (97)[a]	2.3 (1.0)[a]
9	OMe (**2i**)	25 (79)[a]	1.0 (0.8)[a]
10[c]	NH_2 (**2j**)	N.D.	N.A.
11[c]	NMe_2 (**2k**)	N.D.	N.A.

[a] Reaction time is 20 h.

IV. Hydrosilylation of 2a Catalyzed by $Ln[N(SiHMe_2)_2]_3(thf)_2$

In an Ar-filled glove box, to a solution of **4a**, **4b**, or **4f** (0.005 mmol) in benzene (0.25 mL) in a collection vial was added a solution of **2a** (0.100 mmol) in benzene (0.25 mL) and $HN(SiHMe_2)_2$ at ambient temperature. The reaction mixture was stirred for 5 h in a glovebox. After the reaction was finished, the collection vial was quickly removed from a glovebox, and then ether was added for quenching. Internal standard (1,3,5-trimethoxybenzene) was added to the reaction mixture, and all volatiles were removed under reduced pressure. The residue was dissolved in $CDCl_3$, and the yield of $HN[Si(OCHPh_2)Me_2]_2$ was determined by ^{1}H NMR measurement. (The signal intensity of OC*H*Ph_2 was calculated with respect to the internal standard.) The lanthanum complex **1a** showed superior catalytic activity comparable to those of **4a** and **4b**, whereas yield of complexes **1c-f** was almost comparable to the incorporation of all the six Si-H bonds with **2a**, and amine exchange reaction was probably very slow for showing the catalytic performance. For clarifying the reaction intermediates in this lanthanide-catalyzed hydrosilylation, we further investigated the reactivity of ligand-coordinated complexes **4a**, **4b**, and **4f**.

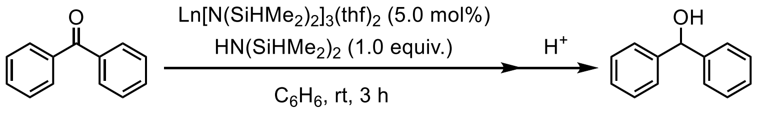

2a **3a**

entry	Ln	Yield of **3a**[b] [%]
1	La (**1a**)	99
2	Ce (**1b**)	58
3	Nd (**1c**)	28
4	Gd (**1d**)	39
5	Lu (**1e**)	32
6	Y (**1f**)	26

^{1}H NMR yield using 1,3,5-trimethoxybenzene as an internal standard.

V. Preparation of Lanthanide Dimethylsilylamide Complexes

V-1. Preparation of Lanthanide Dimethylsilylamide Complexes (**4a**, **4b**, and **4f**)

La(**L**^{1})[N(SiHMe$_2$)$_2$](thf) (**4a**)

To a colorless solution of La[N(SiHMe$_2$)$_2$]$_3$(thf)$_2$ (300.0 mg, 0.441 mmol) in toluene (3 mL), a solution of **L**^{1}H$_2$ (190 mg, 0.437 mmol) in toluene (3 mL) was added. The resulting colorless solution was stirred at ambient temperature for 1 h. All volatiles were removed under reduced pressure, and the residue was washed with hexane (2 mL x 3) to give a white solid. Drying the white powder in vacuo afforded analytically pure **4a** (332.3 mg, 98% yield). Colorless crystals were obtained from the saturated toluene solution at -35 °C. m.p. 148 °C (dec). **^{1}H NMR** (400 MHz, C$_6$D$_6$): δ 0.20 (s, 12H, SiH*Me*$_2$), 1.07 (m, 4H, thf), 1.85 (s, 1H, N*H*), 2.30 (brs, 2H, -NCH$_2$C*H*$_2$-), 2.52 (brs, 2H, -NC*H*$_2$CH$_2$-), 3.35 (m, 4H, thf), 3.61 (brs, 4H, -C*H*$_2$C*H*$_2$N-), 4.75 (s, 2H, Si*H*Me$_2$). **^{19}F NMR** (376 MHz, C$_6$D$_6$): δ -184.2 (brs, 2F, *p*-F), -166.5 (t, $^2J_{FF}$ = 19.0 Hz, 4F, *m*-F), -160.2 (brs, 4F, *o*-F). **^{13}C NMR** (100 MHz, C$_6$D$_6$): δ -141.1 (d, $^1J_{CF}$ = 42 Hz), -138.7 (d, $^1J_{CF}$ = 61 Hz), -134.4, -129.7, 69.8, 51.1, 50.8, 25.2, 2.6. **^{29}Si NMR** (79 MHz, C$_6$D$_6$): δ -27.0 ($^1J_{SiH}$ = 153 Hz). Anal. calcd for C$_{24}$H$_{31}$F$_{10}$LaN$_4$OSi$_2$: C, 37.12; H, 4.02; N, 7.21. Found C, 37.56; H, 3.79; N, 7.20. **IR** (KBr, *v*(SiH), cm^{-1}): 2002 vs

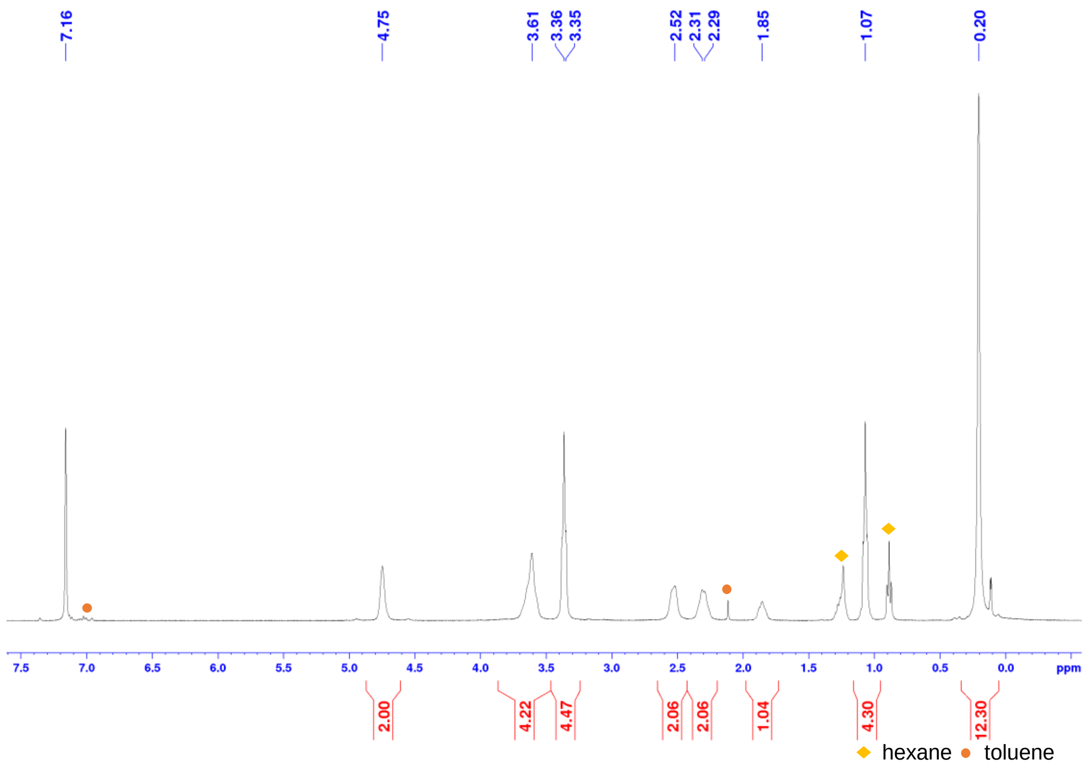

Figure S1. ^{1}H NMR spectrum (400 MHz, C$_6$D$_6$, 30 °C) of complex **4a**.

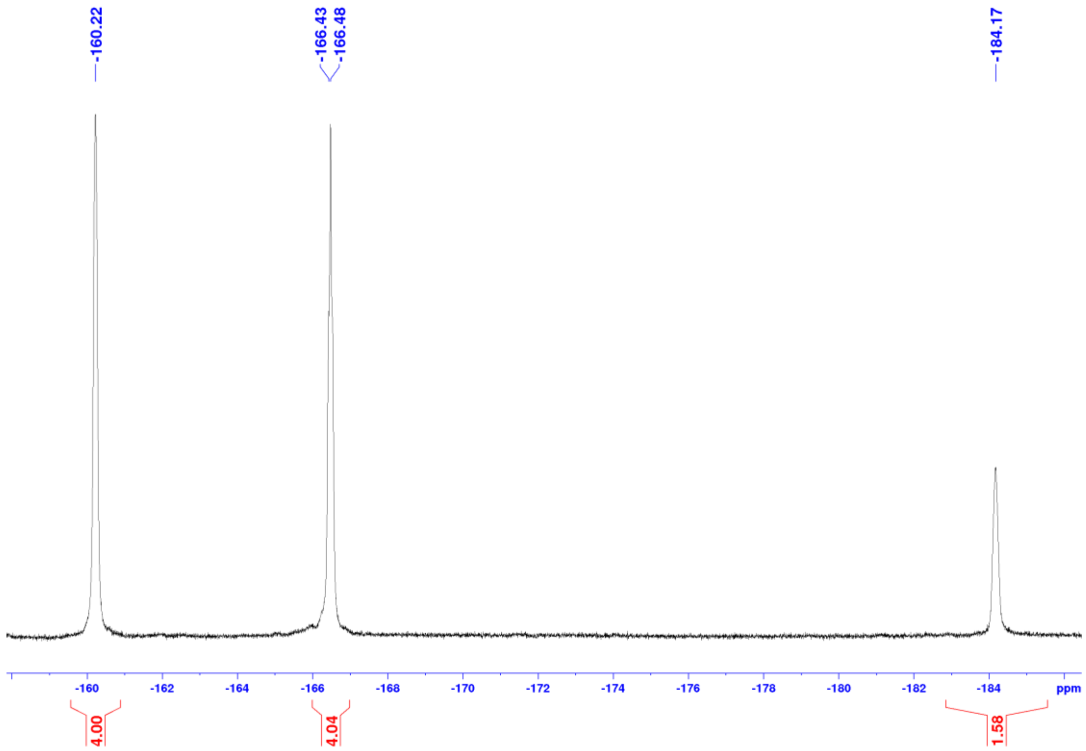

Figure S2. ^{19}F NMR spectrum (376 MHz, C_6D_6, 30 °C) of complex **4a**.

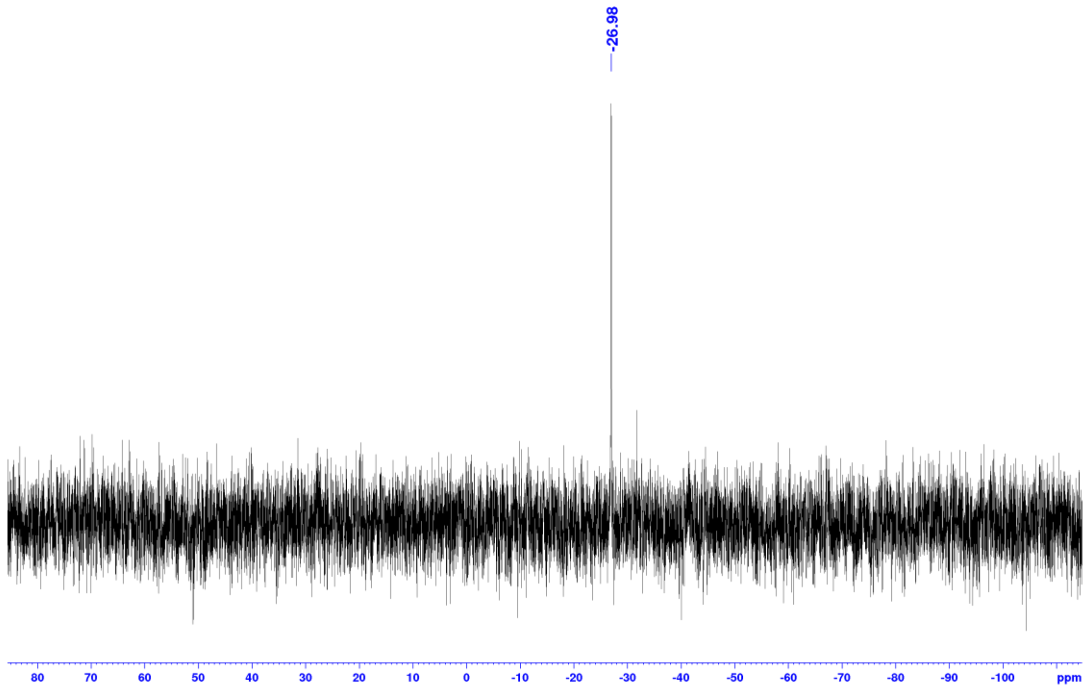

Figure S3. ^{29}Si NMR spectrum (79 MHz, C_6D_6. 30 °C) of complex **4a**.

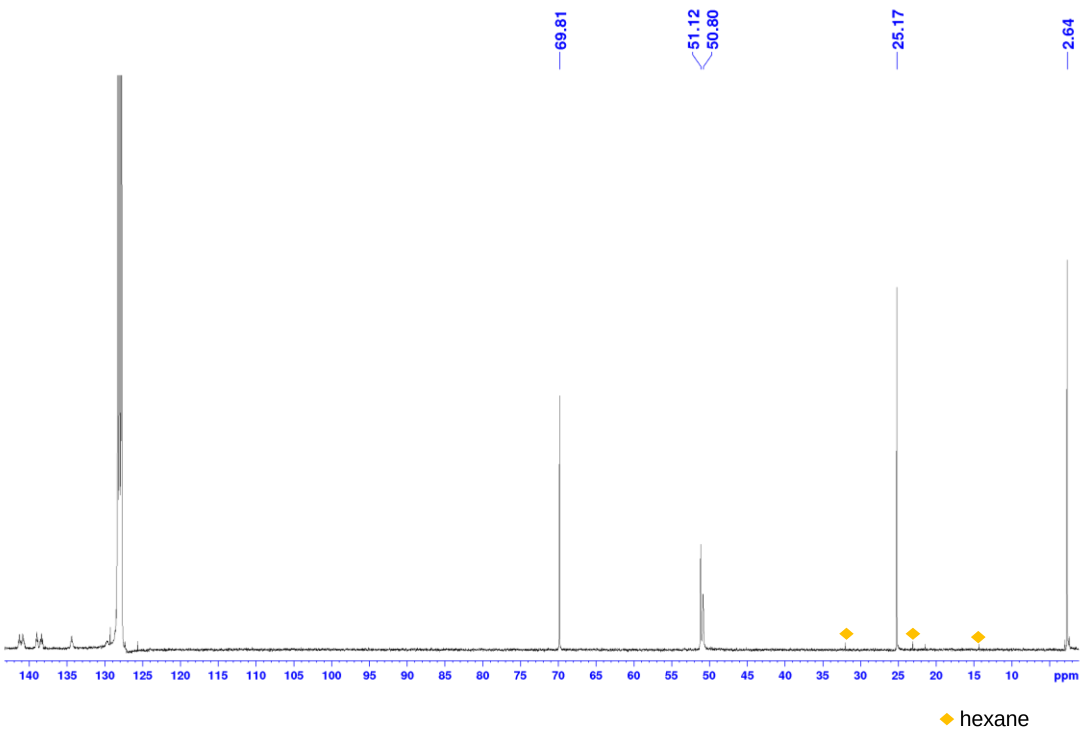

Figure S4. ^{13}C NMR spectrum (100 MHz, C_6D_6, 30 °C) of complex **4a**.

Ce(**L**^{1})[N(SiHMe$_2$)$_2$](thf) (**4b**)

To a colorless solution of Ce[N(SiHMe$_2$)$_2$]$_3$(thf)$_2$ (200.0 mg, 0.294 mmol) in toluene (2 mL), a solution of **L**^{1}H$_2$ (127.8 mg, 0.294 mmol) in toluene (1.5 mL) was added. The resulting colorless solution was stirred at ambient temperature for 1 h. All volatiles were removed under reduced pressure, and the residue was washed with hexane (2 mL x 4) to give white solid. Drying the white powder in vacuo afforded analytically pure **4b** (198.9 mg, 87% yield). Colorless crystals were obtained from the saturated toluene solution at -35 °C. m.p. 125 °C (dec). **^{1}H NMR** (400 MHz, C_6D_6): δ -54.20 (brs, 2H, Si*H*), -19.03 (brs, 1H, N*H*), -8.63 (brs, 4H, -C*H*$_2$-), 2.60 (brs, 12H, Si*Me*$_2$), 11.95 (brs, 4H, -C*H*$_2$-). **^{19}F NMR** (376 MHz, C_6D_6): δ -182.7 (t, J_{FF} = 22.6 Hz, 2F, *p*-F), -178.5 (brs, 4F, *o*-F), -165.6 (d, J_{FF} = 22.6 Hz, 4F, *m*-F). **^{13}C NMR** (100 MHz, C_6D_6): δ -155.1, -146.0, -143.6, -132.8 (d, $^1J_{CF}$ = 235 Hz), 59.0, 42.9, 24.5, 18.8, 7.9. **^{29}Si NMR** (79 MHz, C_6D_6): δ -11.1. Anal. calcd for $C_{24}H_{31}CeF_{10}N_4OSi_2$: C, 37.08; H, 4.02; N, 7.20. Found C, 37.02; H, 4.04; N, 7.12.

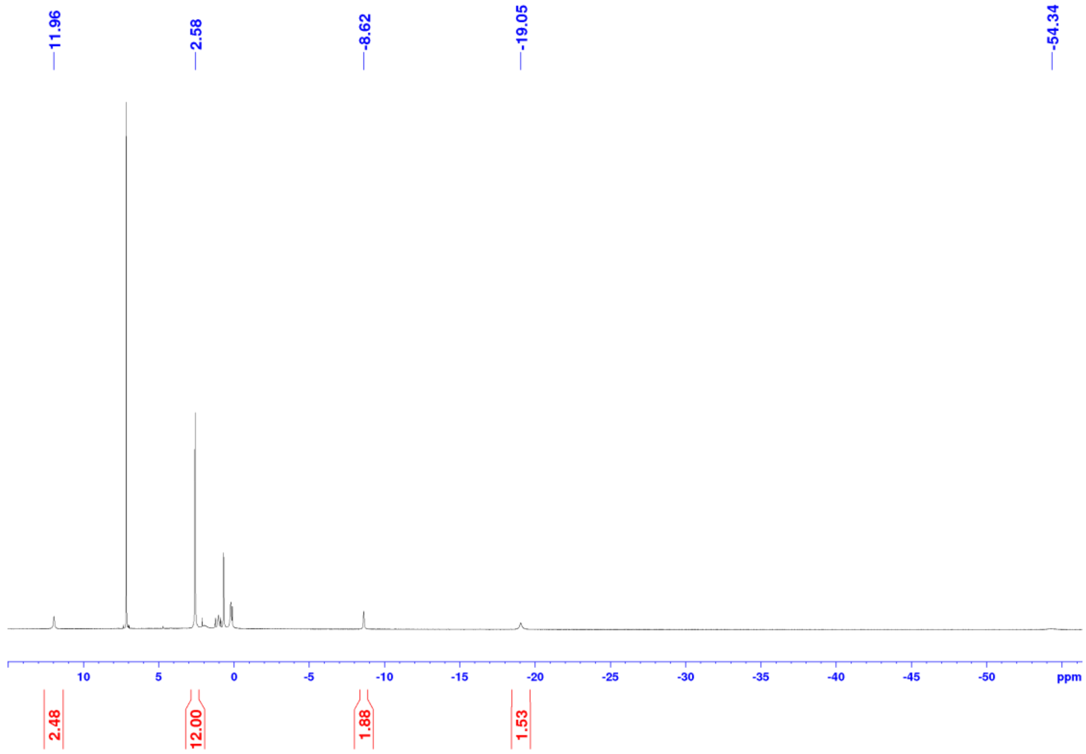

Figure S5. ^{1}H NMR spectrum (400 MHz, C_6D_6, 30 °C) of complex **4b**.

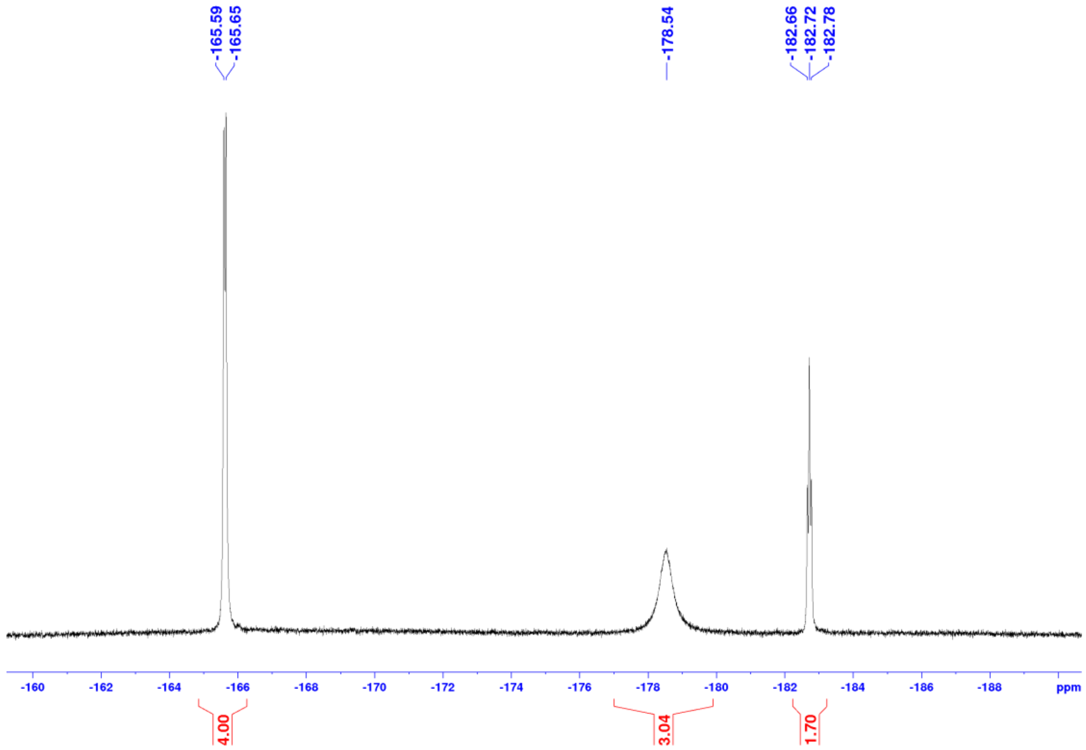

Figure S6. ^{19}F NMR spectrum (376 MHz, C_6D_6, 30 °C) of complex **4b**.

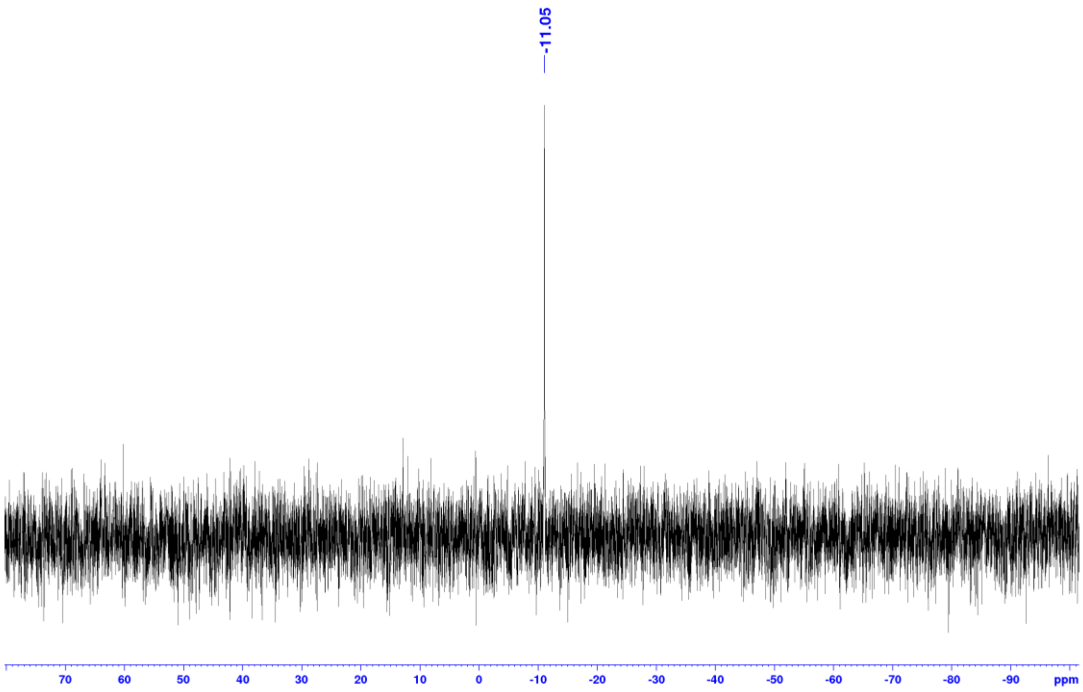

Figure S7. ^{29}Si NMR spectrum (79 MHz, C_6D_6. 30 °C) of complex **4b**.

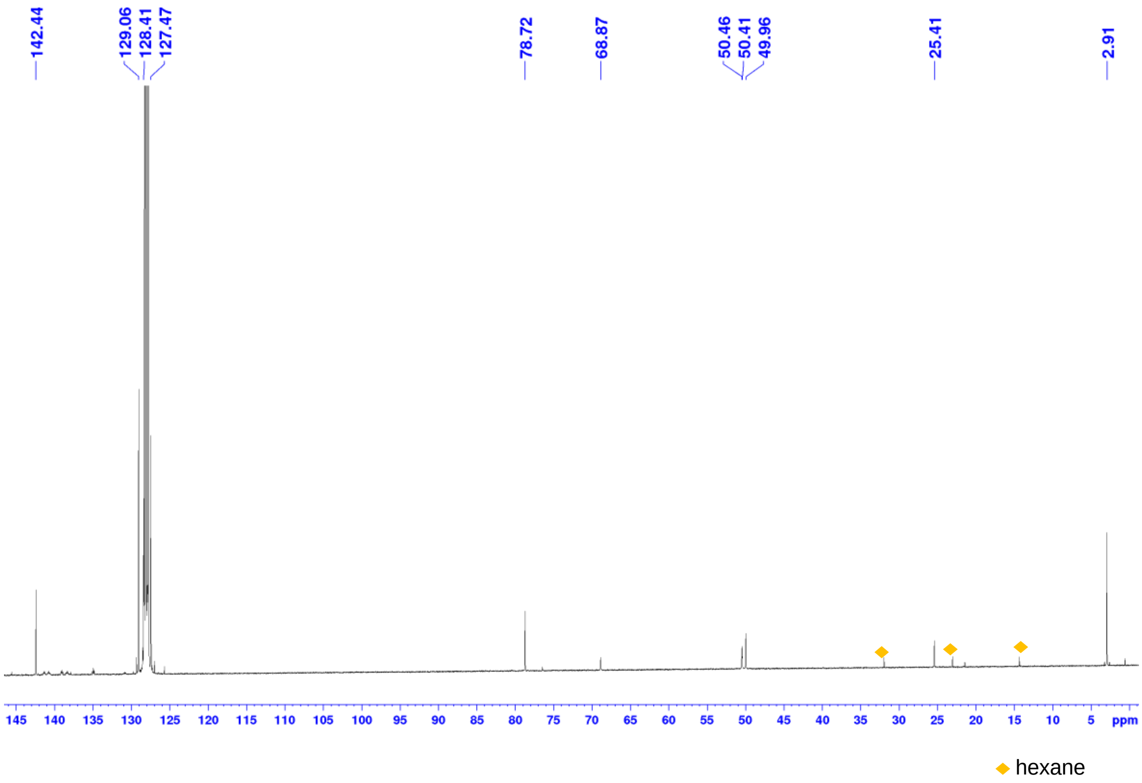

Figure S8. ^{13}C NMR spectrum (100 MHz, C_6D_6, 30 °C) of complex **4b**.

Y(**L**[1])[N(SiHMe$_2$)$_2$](thf) (**4f**)

To a colorless solution of Y[N(SiHMe$_2$)$_2$]$_3$(thf)$_2$ (150.0 mg, 0.238 mmol) in toluene (2 mL), a solution of **L**^{1}H$_2$ (103.6 mg, 0.238 mmol) in toluene (1 mL) was added. The resulting colorless solution was stirred at ambient temperature for 1 h. All volatiles were removed under reduced pressure, and the residue was washed with hexane (2 mL x 2) to give white solid. Drying the white powder in vacuo afforded **4f** (151.0 mg, 87% yield). Colorless crystals were obtained from the saturated toluene solution at -35 °C. m.p. 140 °C (dec). **^{1}H NMR** (400 MHz, C_6D_6): δ 0.18 (d, 12H, SiH*Me*$_2$, 2.9 Hz), 0.98 (m, 4H, thf), 1.98 (s, 1H, N*H*), 2.21 (m, 2H, -NCH$_2$C*H*$_2$-), 2.51 (q, 2H, -NC*H*$_2$CH$_2$-, 4,2 and 11.5 Hz), 3.37 (t, 4H, thf, 12.7 Hz), 3.51 (d, 2H, -C*H*$_2$CH$_2$N-, 6.1 Hz), 3.59 (d, 2H, -C*H*$_2$CH$_2$N-, 12.9 Hz), 4.69 (s, 2H, Si*H*Me$_2$). **^{19}F NMR** (376 MHz, C_6D_6): δ -183.1 (sept, J_{FF} = 11.1, 22.4, and 33.6 Hz, 2F, *p*-F), -166.0 (t, J_{FF} = 22.6 Hz, 4F, *m*-F), -161.4 (s, 4F, *o*-F). **^{13}C NMR** (100 MHz, C_6D_6): δ -141.0 (d, $^1J_{CF}$ = 63 Hz), -138.8 (d, $^1J_{CF}$ = 87 Hz), -134.1, -130.1, 70.7, 50.0, 49.4, 24.9, 3.1. **^{29}Si NMR** (79 MHz, C_6D_6): δ -23.9 ($^1J_{SiH}$ = 164 Hz). Anal. calcd for $C_{24}H_{31}F_{10}N_4OSi_2Y$: C, 39.67; H, 4.30; N, 7.71. Found C, 43.72; H, 4.55; N, 6.99. The carbon and nitrogen contents in the elemental analysis was out of range, even though the microcrystals were used. **IR** (KBr, *ν*(SiH), cm^{-1}): 2017 br, 2051 m

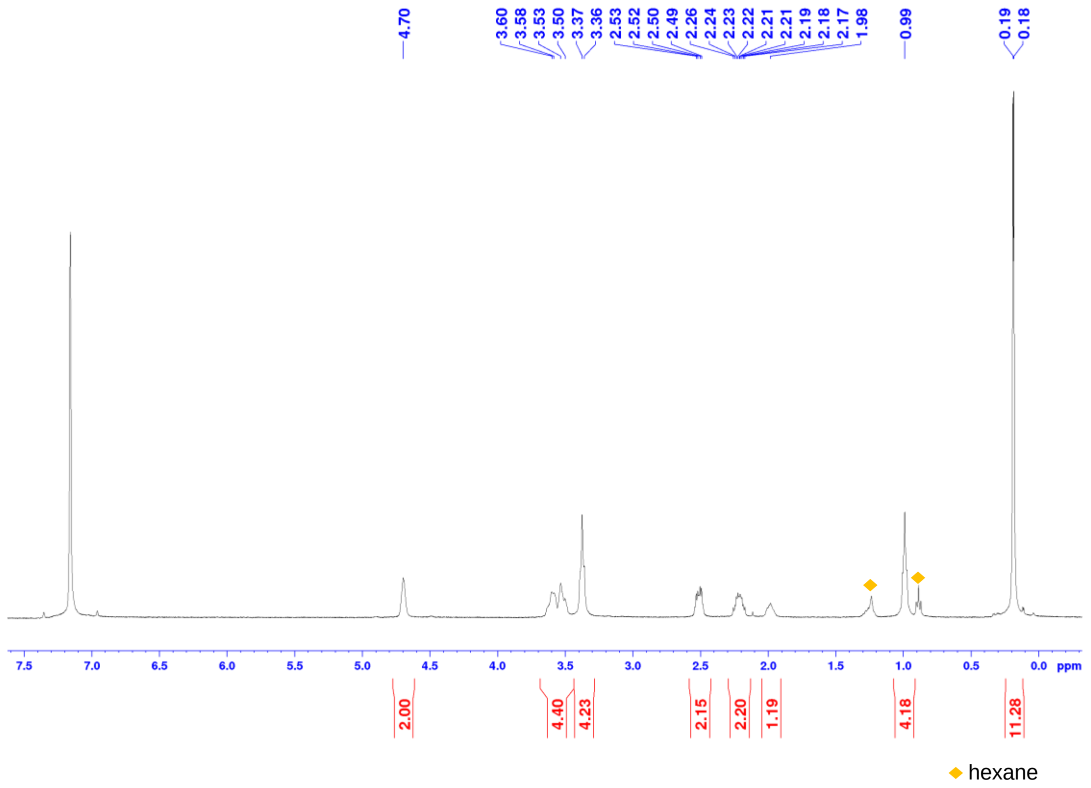

Figure S9. ^{1}H NMR spectrum (400 MHz, C_6D_6, 30 °C) of complex **4f**.

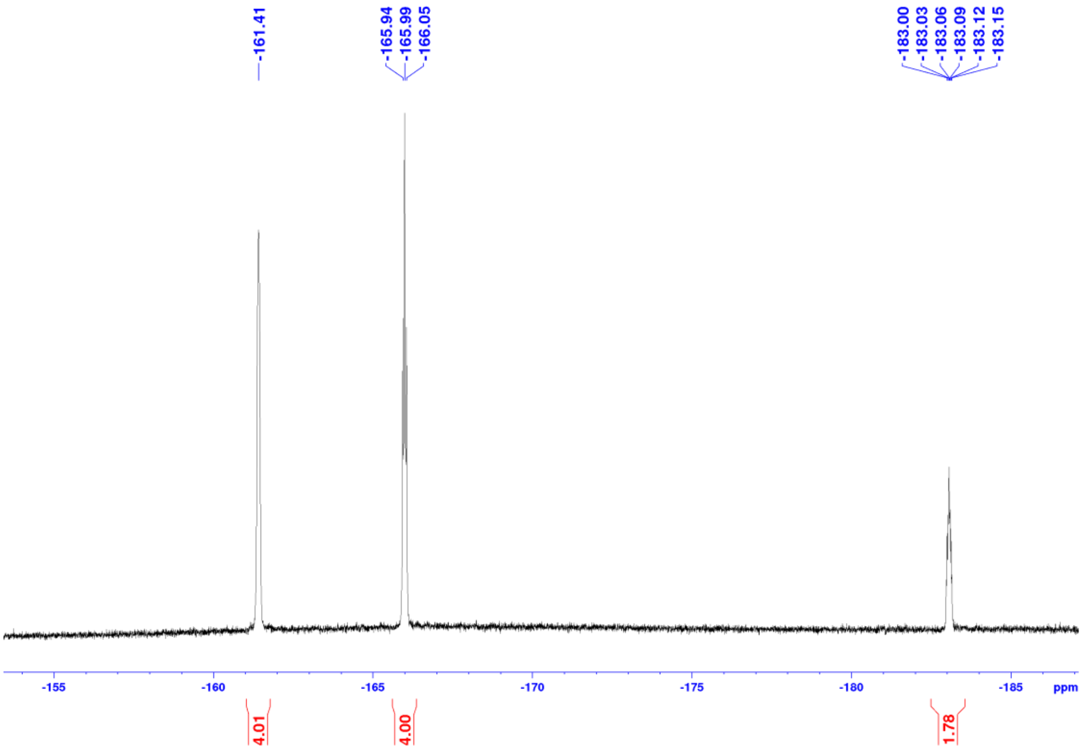

Figure S10. ^{19}F NMR spectrum (376 MHz, C_6D_6, 30 °C) of complex **4f**.

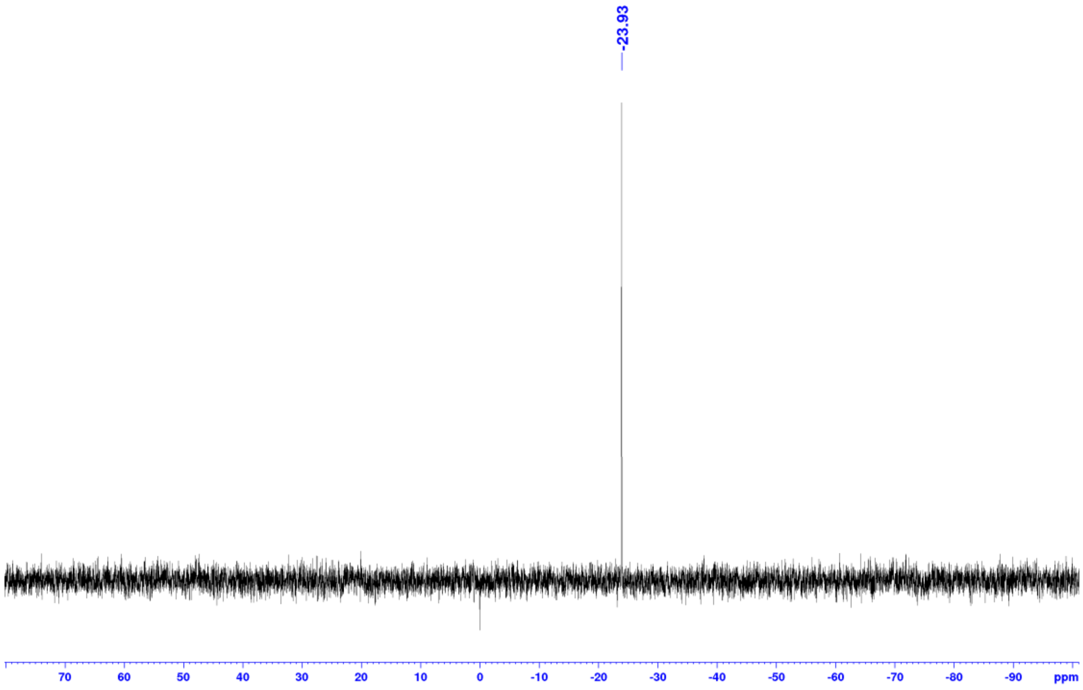

Figure S11. ^{29}Si NMR spectrum (79 MHz, C_6D_6. 30 °C) of complex **4f**.

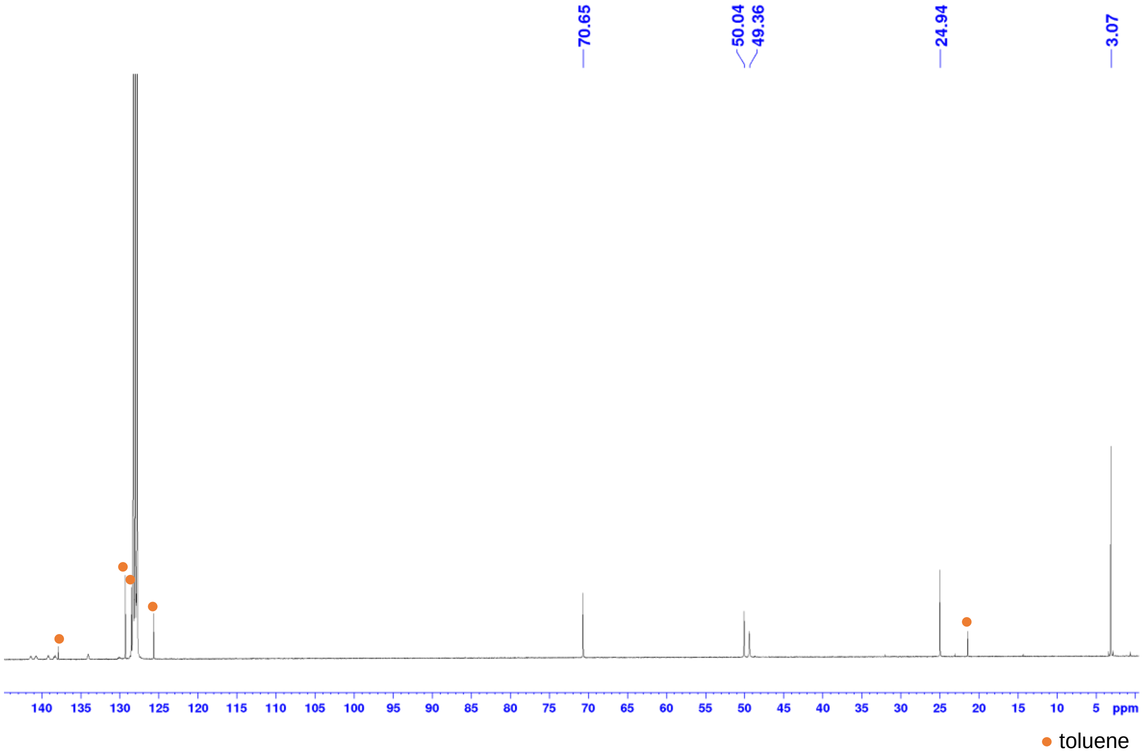

Figure S12. ^{13}C NMR spectrum (100 MHz, C_6D_6, 30 °C) of complex **4f**.

V-2. Preparation of Doubly Hydrosilylated Complexes (**5aa**, **5ba,** and **5fa**)

La(**L**^{1})[N[Si(OCHPh$_2$)Me$_2$]$_2$](thf) (**5aa**)

To a colorless solution of **4a** (30.6 mg, 0.0386 mmol) in toluene (0.7 mL), a solution of **2a** (13.7 mg, 0.0753 mmol) in toluene (0.3 mL) was added. The color of reaction mixture immediately changed to red. After stirring for 1 h at ambient temperature, a resulting colorless solution was dried under reduced pressure to give **5aa** as pale blue powder (38.5 mg, 90% yield). Colorless crystals were obtained from the saturated toluene solution at -35 °C. m.p. 42 °C (dec). **^{1}H NMR** (400 MHz, C_6D_6): δ 0.14 (s, 12H, SiH*Me*$_2$), 0.53 (brs, 1H, N*H*), 1.27 (brs, 4H, thf), 1.91 (m, 2H, -NCH$_2$C*H*$_2$-), 2.03 (brs, 2H, -NC*H*$_2$CH$_2$-), 3.14 (brs, 2H, -C*H*$_2$CH$_2$N-), 3.37 (d, 2H, -C*H*$_2$CH$_2$N-, 13.8 Hz), 3.40 (brs, 2H, thf), 5.83 (s, 2H, Si*H*Me$_2$), 6.92 (t, 4H, *Ar*, 7.1 Hz), 7.02 (t, 8H, *Ar*, 7.4 Hz), 7.17 (d, 8H, *Ar*, 7.6 Hz). **^{19}F NMR** (376 MHz, C_6D_6): δ -182.2 (sept, J_{FF} = 11.0, 21.7, and 32.6 Hz, 2F, *p*-F), -166.4 (t, J_{FF} = 21.9 Hz, 4F, *m*-F), -156.2 (dd, J_{FF} = 10.2 and 19.9 Hz, 4F, *o*-F). **^{13}C NMR** (100 MHz, C_6D_6) δ 142.4, 141.1 (d, $^{1}J_{CF}$ = 54 Hz), 138.7 (d, $^{1}J_{CF}$ = 73 Hz), 135.0, 130.9, 129.1, 128.4, 127.5, 78.7, 68.9, 50.5, 50.0, 25.4, 2.9. The ^{29}Si NMR signal of the silylamide moiety was not observed under these conditions due to low intensity of this signal. Anal. calcd for $C_{50}H_{51}F_{10}LaN_4O_3Si_2$: C, 52.63; H, 4.51; N, 4.91. Found C, 52.19; H, 3.99; N, 5.09.

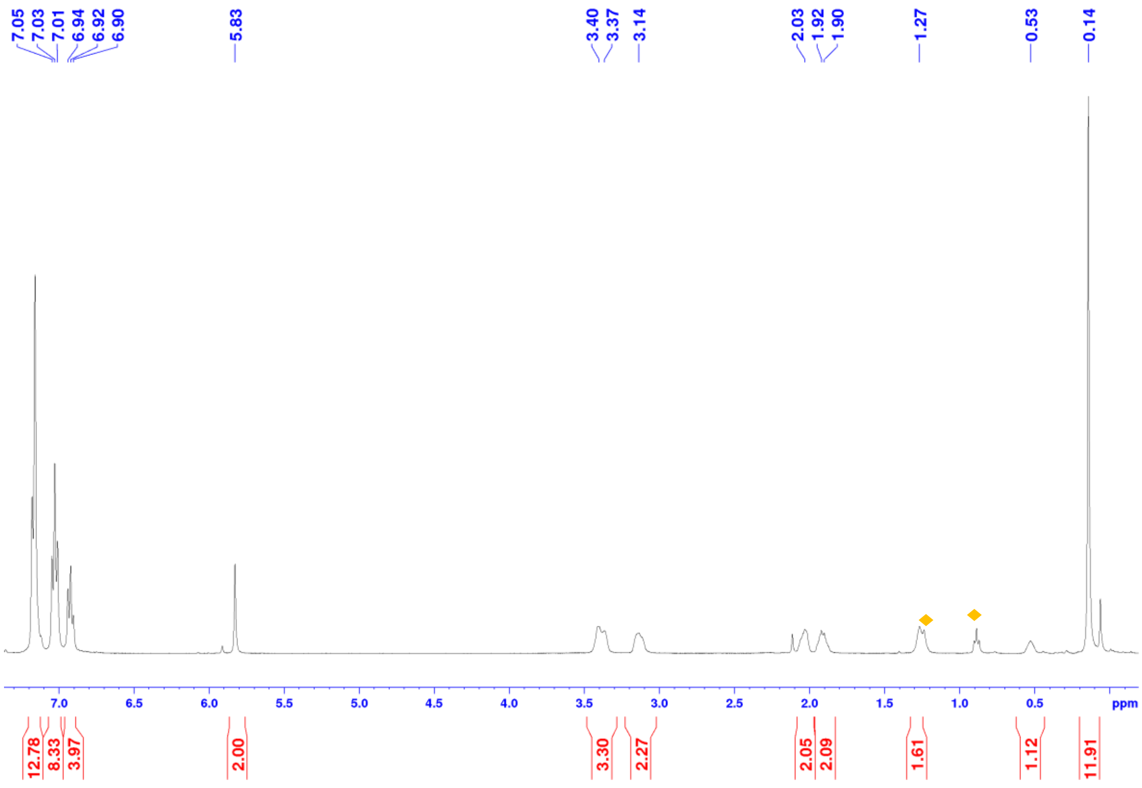

Figure S13. ^{1}H NMR spectrum (400 MHz, C_6D_6, 30 °C) of complex **5aa**.

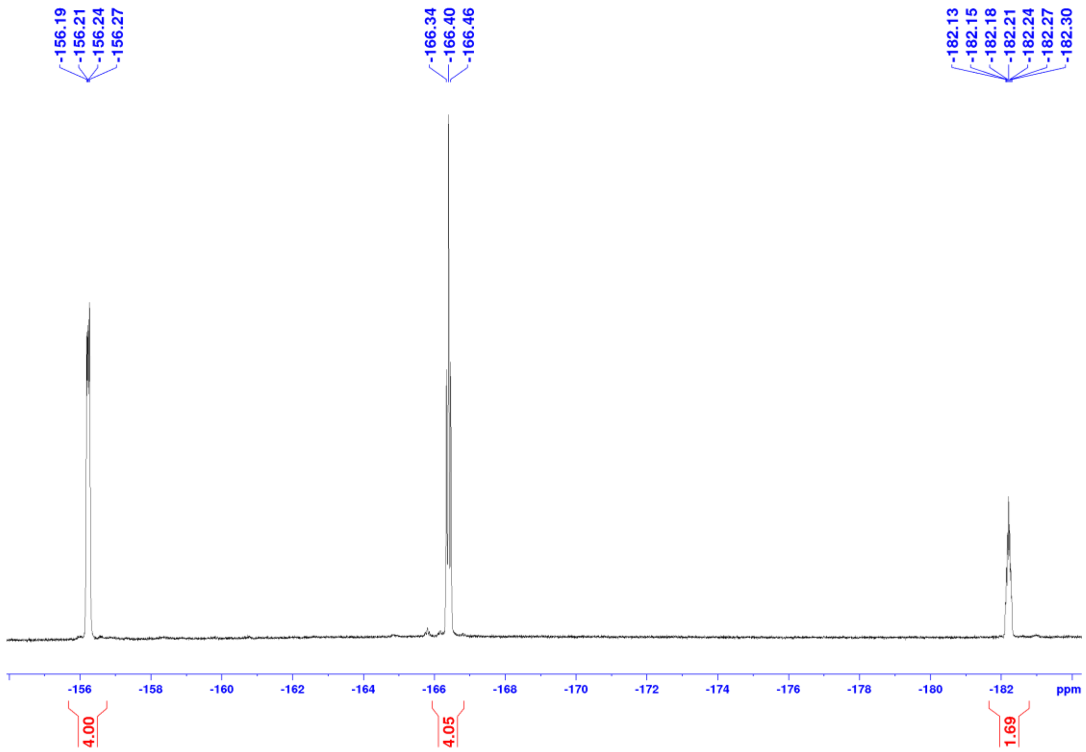

Figure S14. ^{19}F NMR spectrum (376 MHz, C_6D_6, 30 °C) of complex **5aa**.

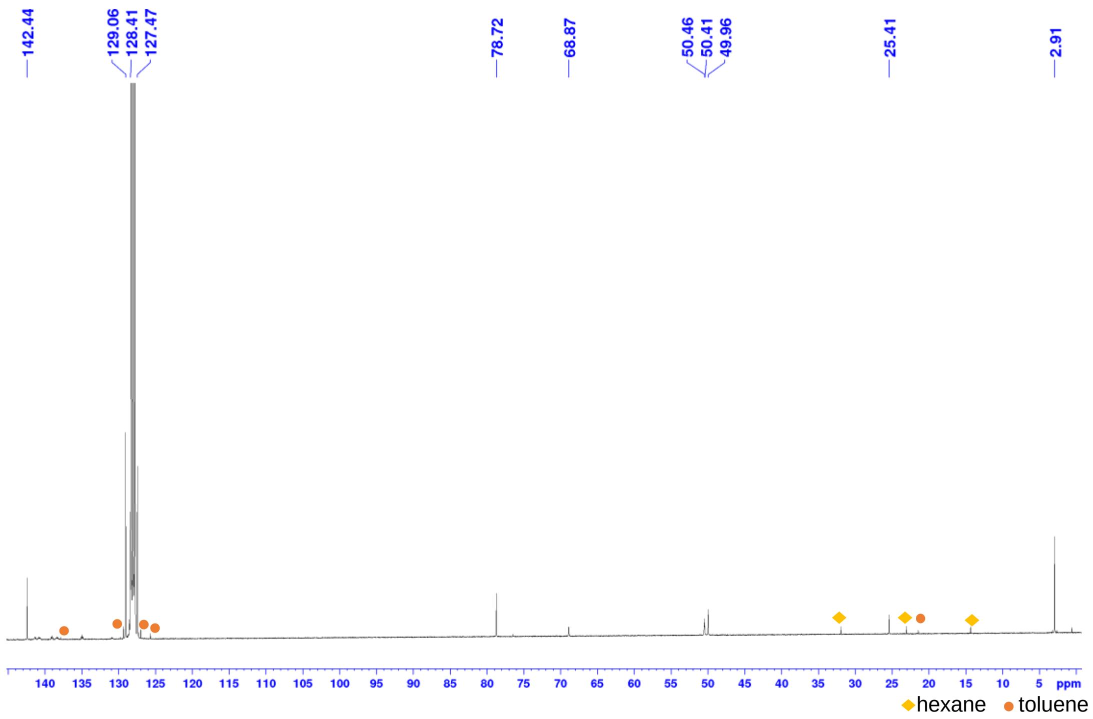

Figure S15. ^{13}C NMR spectrum (100 MHz, C_6D_6, 30 °C) of complex **5aa**.

Ce(**L**[1])[N[Si(OCHPh$_2$)Me$_2$]$_2$](thf) (**5ba**)

To a colorless solution of **4b** (80.0 mg, 0.103 mmol) in C_6H_6 (2 mL), a solution of **2a** (37.5 mg, 0.206 mmol) in C_6H_6 (2 mL) was added. The color of reaction mixture immediately changed to red. After stirring for 1 h at ambient temperature, a resulting pale red solution was dried under reduced pressure to give **5ba** as pale red powder (61.2 mg, 52% yield). Colorless crystals were obtained from the saturated toluene solution at -35 °C. m.p. 43 °C (dec). **^{1}H NMR** (400 MHz, C_6D_6): δ -4.07, -3.55, 1.80, 3.58, 4.35, 4.51, 5.36, 14.34, 17.98, 18.15. **^{19}F NMR** (376 MHz, C_6D_6): δ -181.6 (t, J_{FF} = 22.5 Hz, 2F, *para*), -177.5 (brs, 4F, *ortho*), -166.1 (d, J_{FF} = 22.6 Hz, 4F, *meta*). **^{13}C NMR** (100 MHz, C_6D_6): δ 142.4, 140.0, 132.3, 130.0, 128.7, 124.9, 124.1, 116.7, 67.1, 59.0, 57.2, 51.9, 26.7, 10.3. The ^{29}Si NMR signal of the silylamide moiety was not observed under these conditions due to low intensity of this signal. Anal. calcd for $C_{50}H_{51}CeF_{10}N_4O_3Si_2$: C, 52.58; H, 4.50; N, 4.91. Found C, 52.01; H, 4.30; N, 5.08.

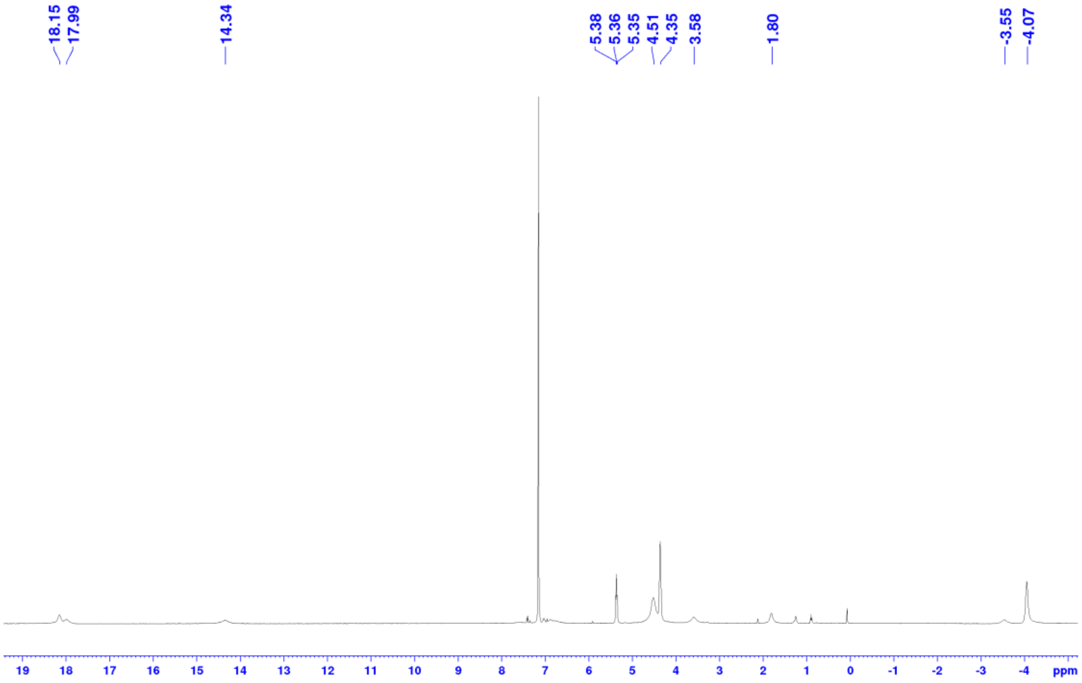

Figure S16. ^{1}H NMR spectrum (400 MHz, C_6D_6, 30 °C) of complex **5ba**.

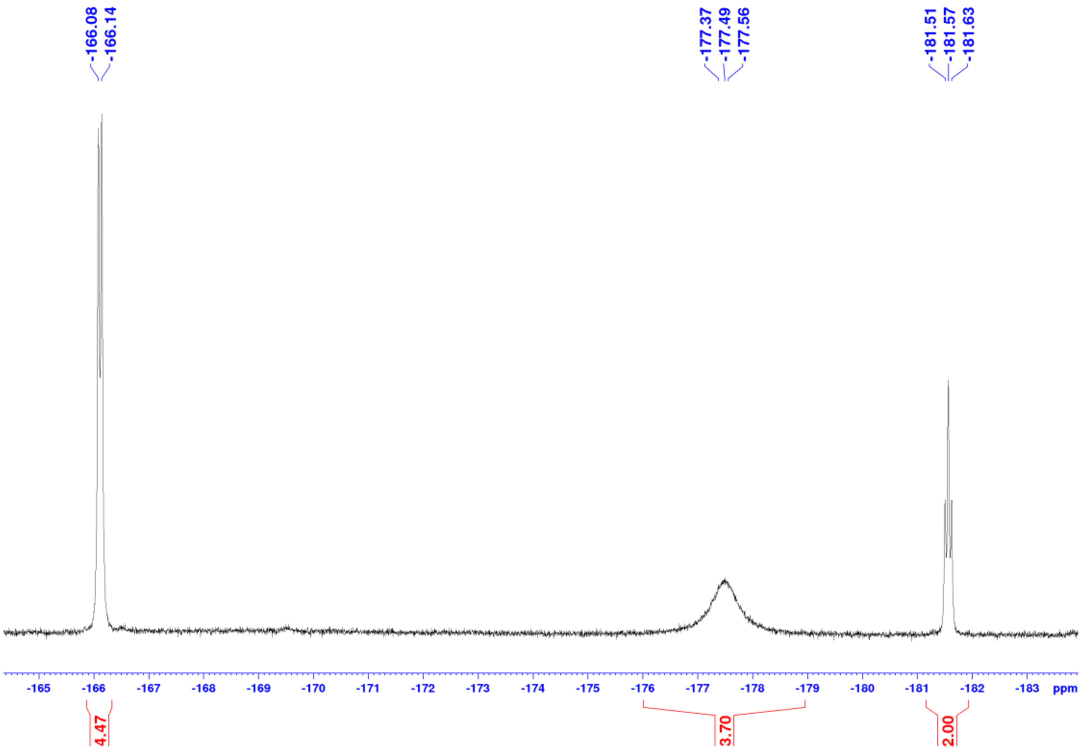

Figure S17. ^{19}F NMR spectrum (376 MHz, C_6D_6, 30 °C) of complex **5ba**.

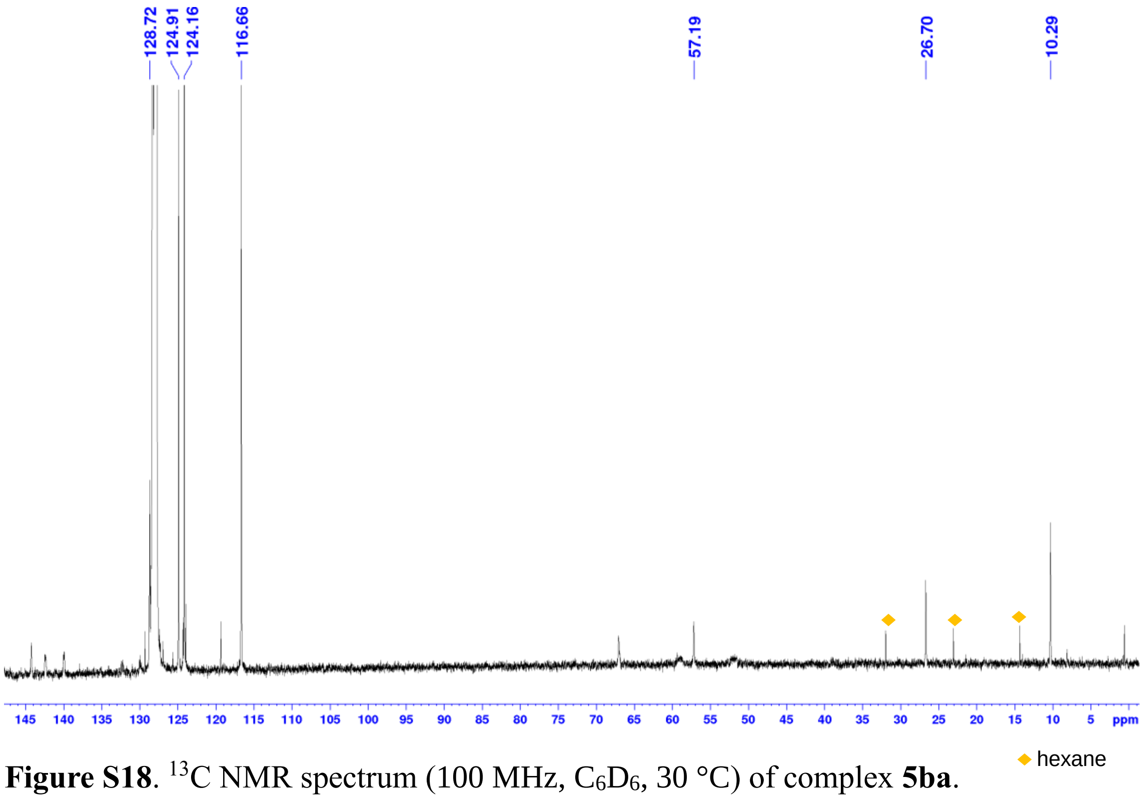

Figure S18. ^{13}C NMR spectrum (100 MHz, C_6D_6, 30 °C) of complex **5ba**.

Y(**L**^{1})[N[Si(OCHPh$_2$)Me$_2$]$_2$] (**5fa**)

To a colorless solution of **4f** (100.0 mg, 0.137 mmol) in toluene (3 mL), a solution of **2a** (49.4 mg, 0.271 mmol) in toluene (1 mL) was added. The color of reaction mixture immediately changed to red. After stirring for 1 h at ambient temperature, a resulting pale red solution was dried under reduced pressure to give **5fa** as a pale red powder (114.4 mg, 77% yield). m.p. 45 °C (dec). **^{1}H NMR** (400 MHz, C_6D_6): δ 0.03 (s, 12H, Si*Me*$_2$), 2.35 (m, 2H, C*H*$_2$), 2.52 (brs, 2H, C*H*$_2$), 3.41 (t, 2H, C*H*$_2$, 5.2 Hz), 3.51 (brs, 2H, C*H*$_2$), 4.47 (brs, 1H, N*H*), 5.83 (s, 2H, OC*H*Ph$_2$), 7.03 (t, 4H, *p-H*, 7.2 Hz), 7.13 (t, 8H, *m-H*, 7.5 Hz), 7.21 (d, 8H, *o-H*, 7.5 Hz). **^{19}F NMR** (376 MHz, C_6D_6): δ -183.7 (sept, J_{FF} = 10.2, 22.6, and 33.1 Hz, 2F, *p*-F), -165.2 (t, J_{FF} = 20.6 Hz, 4F, *m*-F), -160.7 (brs, 4F, *o*-F). **^{13}C NMR** (100 MHz, C_6D_6): δ 142.3, 140.3 (d, $^1J_{CF}$ = 59 Hz), 138.1, 133.5, 130.3, 128.7, 128.2, 127.6, 78.1, 47.6, 45.7, 3.0. The ^{29}Si NMR signal of the silylamide moiety was not observed under these conditions due to low intensity of this signal. Anal. calcd for $C_{46}H_{63}F_{10}N_4O_2Si_2Y$: C, 54.22; H, 4.25; N, 5.50. Found C, 59.65; H, 4.35; N, 4.61. The carbon and nitrogen contents in the elemental analysis was out of range, even though the microcrystals were used.

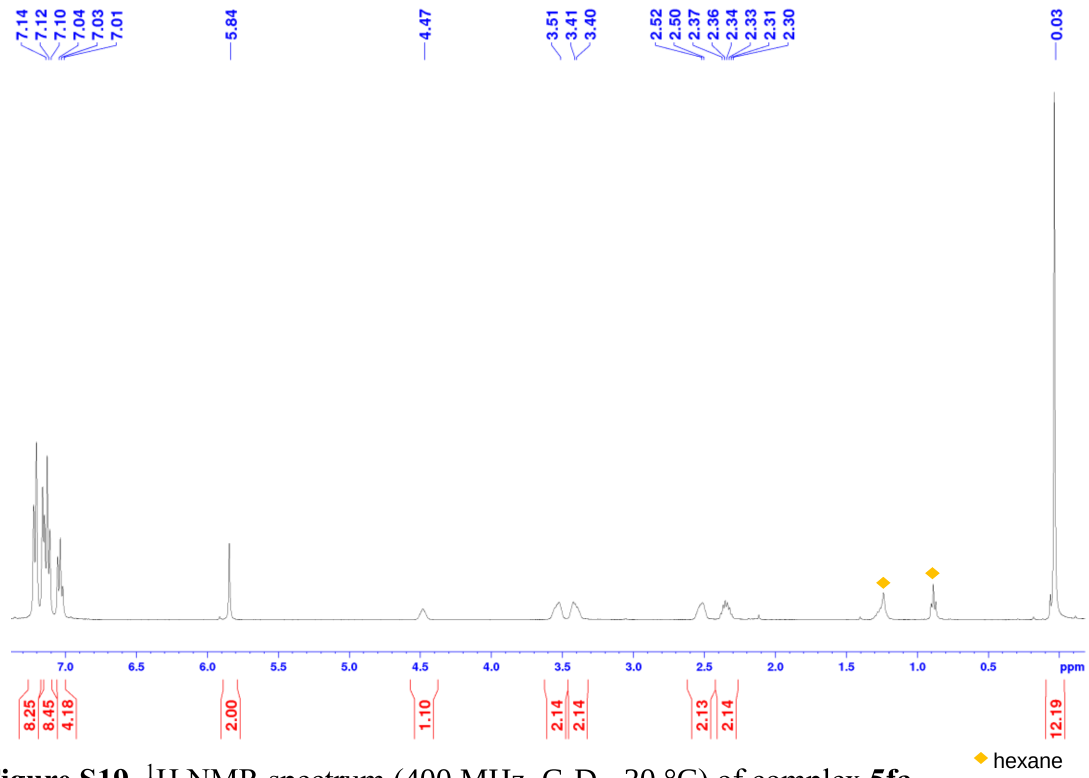

Figure S19. ^{1}H NMR spectrum (400 MHz, C_6D_6, 30 °C) of complex **5fa**.

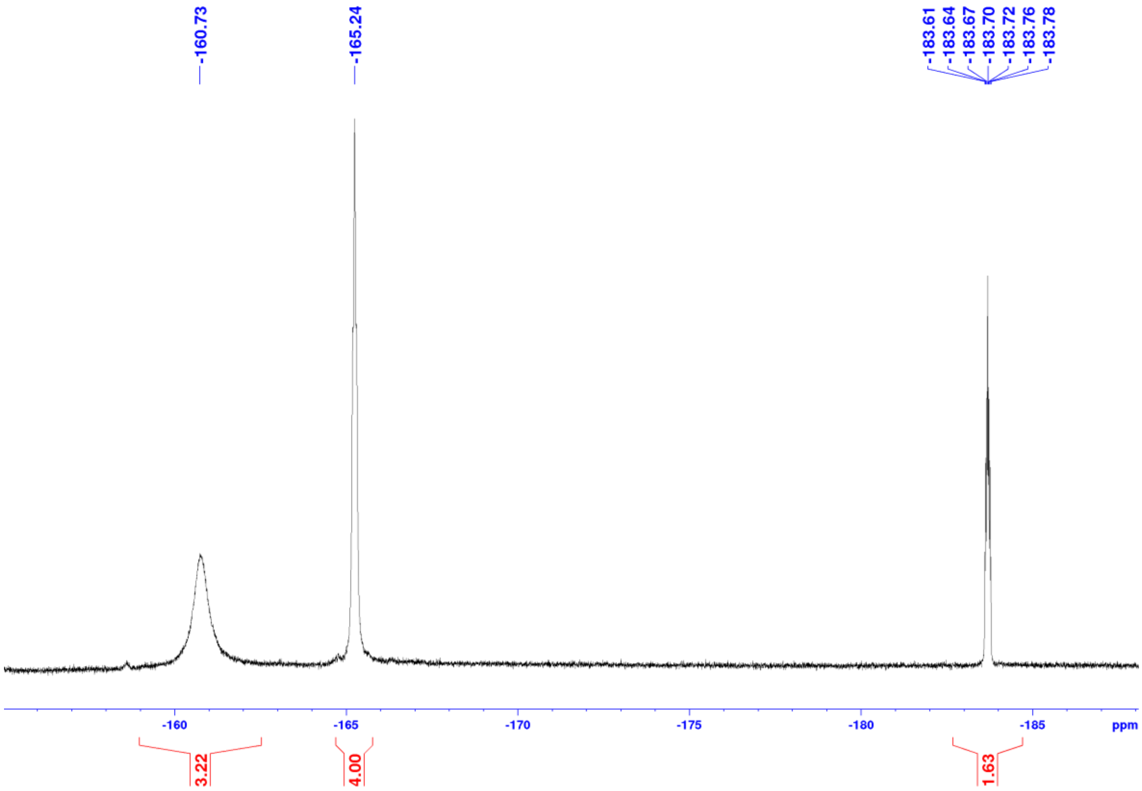

Figure S20. ^{19}F NMR spectrum (376 MHz, C_6D_6, 30 °C) of complex **5fa**.

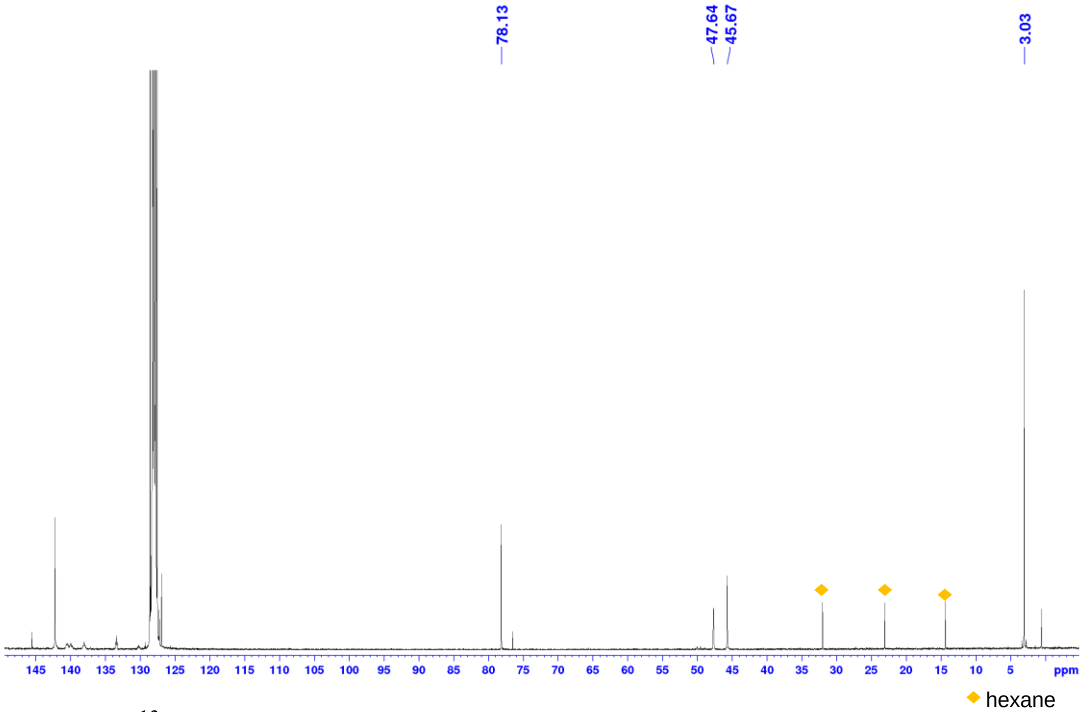

Figure S21. ^{13}C NMR spectrum (100 MHz, C_6D_6, 30 °C) of complex **5fa**.

VI. VT-^{19}F NMR Spectra of 4b in toluene-d_8

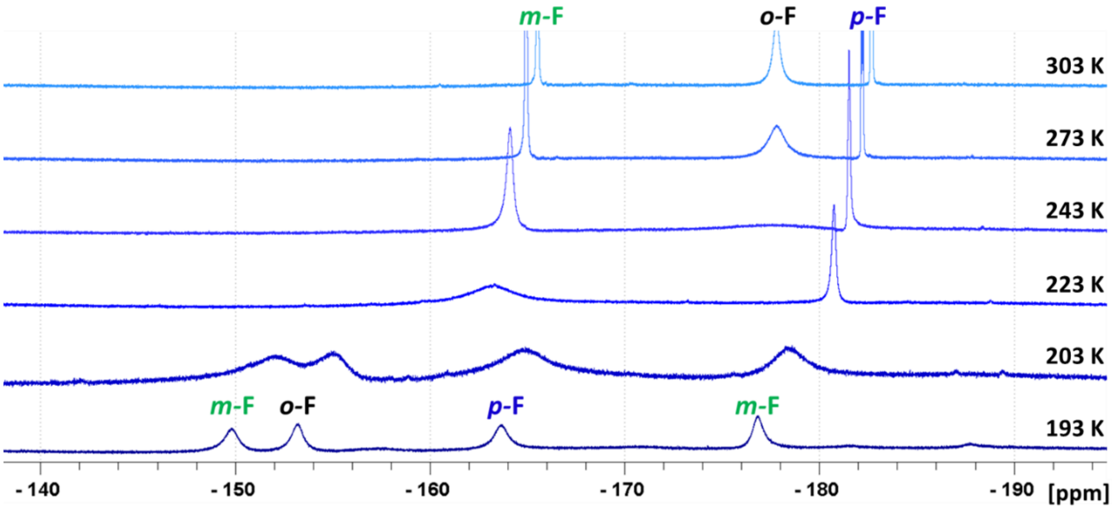

$$\Delta G^{\ddagger} = RT\mathit{ln}\left(\frac{kT}{k_c h}\right), \qquad k_c = \frac{\pi}{\sqrt{2}}\Delta\nu_{AB}$$

$$\Delta G^{\ddagger}_{193} = 4.6\ \ \mathrm{kcalmol^{-1}}$$

Figure S22. VT-^{19}F NMR spectra for **4b** in toluene-d_8 between 193 K—303 K.

VII. Hydrosilylation of 2a Catalyzed by 4a, 4b, and 4f

In an Ar-filled glove box, to a solution of **4a**, **4b**, or **4f** (0.005 mmol) in benzene (0.25 mL) in a collection vial was added a solution of **2a** (0.100 mmol) in benzene (0.25 mL) and $HN(SiHMe_2)_2$ at ambient temperature. The reaction mixture was stirred for 5 h in a glovebox. After the reaction was finished, the collection vial was quickly removed from a glovebox, and then ether was added for quenching. Internal standard (1,3,5-trimethoxybenzene) was added to the reaction mixture, and all volatiles were removed under reduced pressure. The residue was dissolved in $CDCl_3$, and the yield of $HN[Si(OCHPh_2)Me_2]_2$ was determined by ^{1}H NMR measurement. (The signal intensity of OC*H*Ph_2 was calculated with respect to the internal standard.)

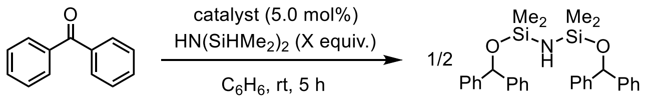

2a

entry	catalyst	X	Yield[b] [%]
1	**4a**	1.0	>99
2	**4b**	1.0	>99
3	**4b**	0.5	46
4	**4f**	1.0	9

^{1}H NMR yield using 1,3,5-trimethoxybenzene as an internal standard.

VIII. Time Course Profiles for Formation of Benzophenone-inserted Complexes 5ah and 5fh

In an Ar-filled glove box, to a solution of **4a** (6.43×10^{-3} mmol) in toluene-d_8 (0.50 mL) placed in a NMR tube was added a solution of **2h** (1.25×10^{-2} mmol) in toluene-d_8 (0.50 mL) at -35 °C. The reaction mixture was quickly removed from the glovebox and cooled down to -78 °C to stop the reaction before ^{1}H NMR measurements. ^{1}H NMR measurements were carried out at 265 K, and the ratio of **4a**, **5ah**, and **6ah** was determined by the signal intensity of $(\mathrm{Si}Me_2)_2$ in **4a**, $(\mathrm{Si}Me_2)_2$ in **5ah**, and $\mathrm{Si}Me_2$ in **6ah**.

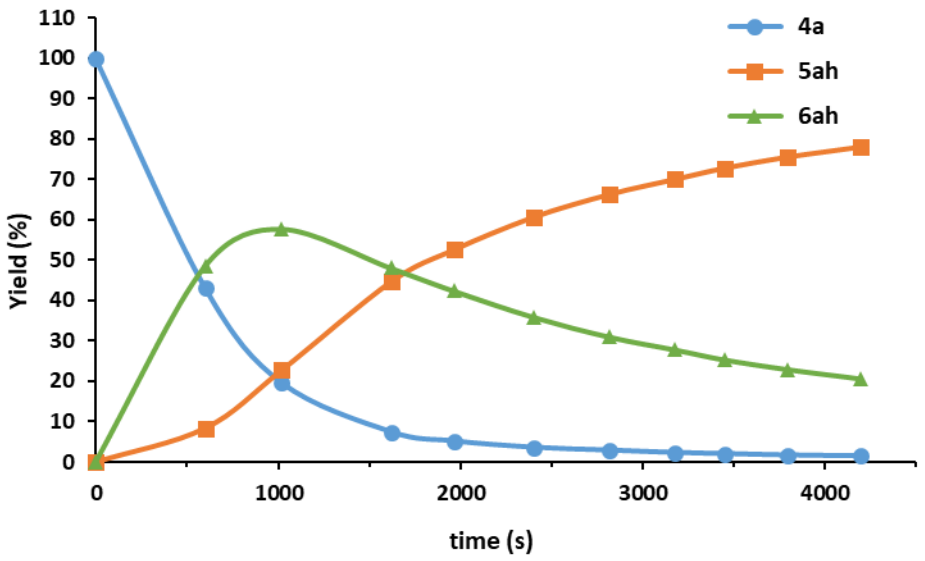

Figure S23. Reaction profile for formation of **5ah** using **4a** and 2 equiv of **2h** in toluene-d_8.

In an Ar-filled glove box, to a solution of **4f** (6.88×10^{-3} mmol) in toluene-d_8 (0.50 mL) placed in a NMR tube was added a solution of **2h** (1.37×10^{-2} mmol) in toluene-d_8 (0.50 mL) at -35 °C. The reaction mixture was quickly removed from the glovebox and cooled down to -78 °C to stop the reaction before ^{1}H NMR measurements. ^{1}H NMR measurements were carried out at 265 K and the ratio of **4f**, **5fh**, and **6fh** was determined by the signal intensity of $(SiMe_2\mathit{H})_2$ in **4a**, $(OC\mathit{H}Ar_2)_2$ in **5fh** and $OC\mathit{H}Ar_2$ in **6fh**.

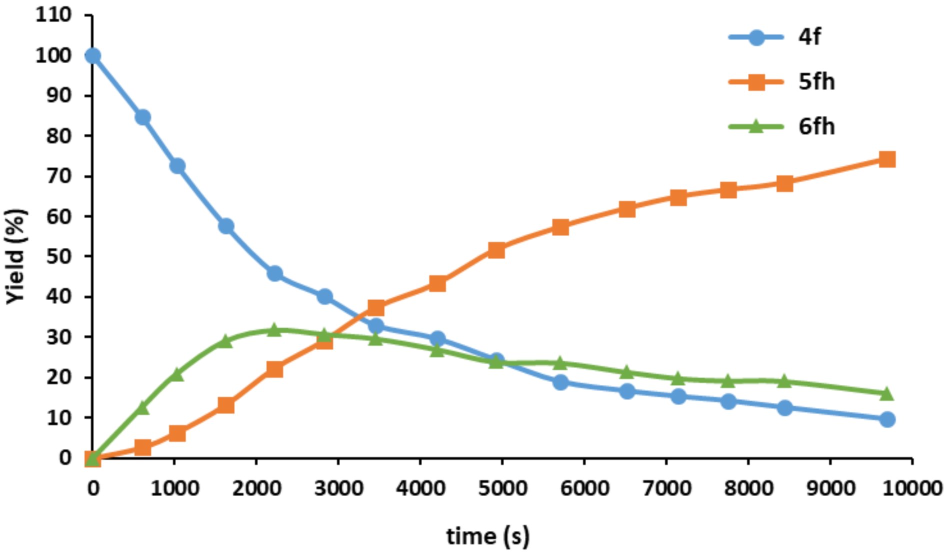

Figure S24. Reaction profile for formation of **5fh** using **4a** and 2 equiv of **2h** in toluene-d_8.

IX. Reaction of 4a and 4f with 1 equiv of 2a

In an Ar-filled glove box, to a solution of **4a** (1.28 x 10^{-2} mmol) in C_6D_6 (0.25 mL) was added a solution of **2a** (1.28 x 10^{-2} mmol) in C_6D_6 (0.25 mL) at ambient temperature. The color of the reaction mixture immediately changed from colorless to red, and returned to colorless after a few seconds. ^{1}H NMR measurements was carried out at ambient temperature to determine the ratio of a double hydrosilylated complex **5aa**, a monohydrosilylated complex **6aa**, and starting complex **4a** based on the signal intensity of $(SiMe_2)_2$ in **4a**, $(OCHPh_2)_2$ in **5aa** and $(SiMe_2)_2$ in **6aa**. Same reaction was conducted by using **4f** instead of **4a**.

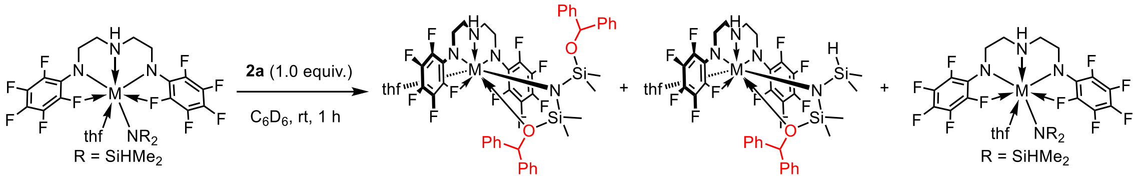

4a: M = La
4f: M = Y

5aa: M = La, n = 1, 38% yield
5fa: M = Y, n = 0, 56% yield

6aa: 40% yield
6fa: 28% yield

4a : 22% yield
4f : 16% yield

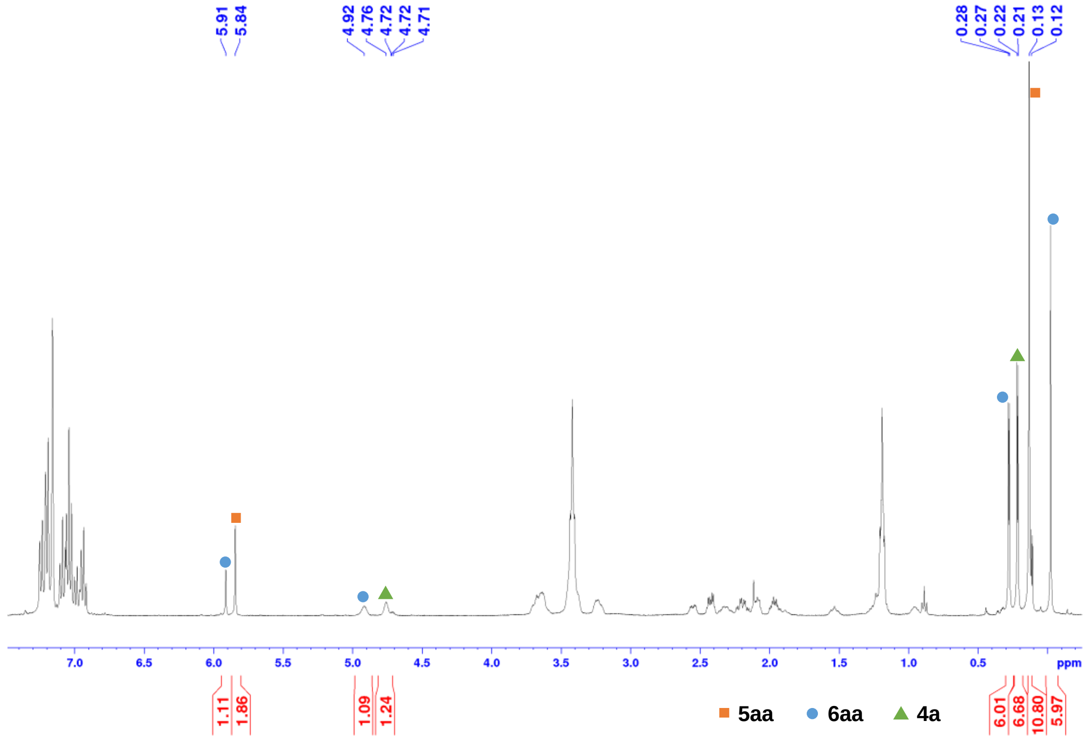

Figure S25. ^{1}H NMR spectrum (400 MHz, C_6D_6, 30 °C) of the reaction mixture of **4a** and 1 equiv of **2a**.

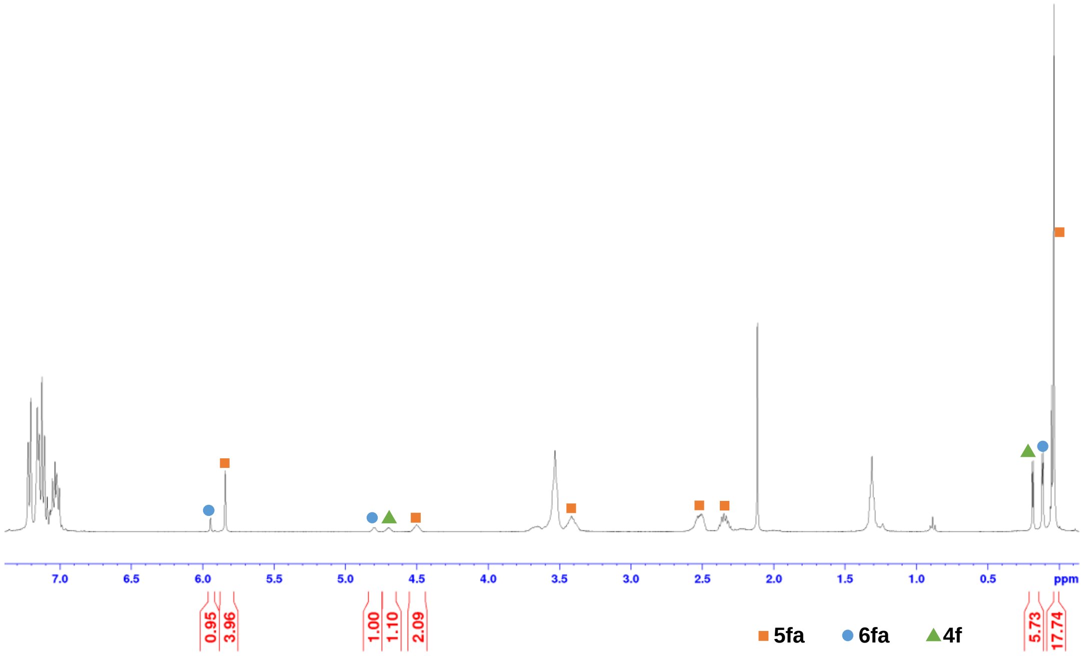

Figure S26. ^{1}H NMR spectrum (400 MHz, C_6D_6, 30 °C) of the reaction mixture of **4f** and 1 equiv of **2a**.

X. Reaction of Double Hydrosilylated Complexes 5aa and 5fa with 1 equiv of 2a

To a solution of **5aa** (4.38×10^{-3} mmol) in C_6D_6 (0.25 mL) was added a solution of **2a** (4.38×10^{-3} mmol) in C_6D_6 (0.25 mL) at room temperature. The color of reaction mixture immediately changed from colorless to red. ^{1}H NMR spectrum for reaction mixture is shown in Figure S27. ^{1}H NMR spectrum for reaction mixture of complex **5fa** and 1 equiv of **2a** indicated no interaction between **5fa** and **2a** (Figure S28).

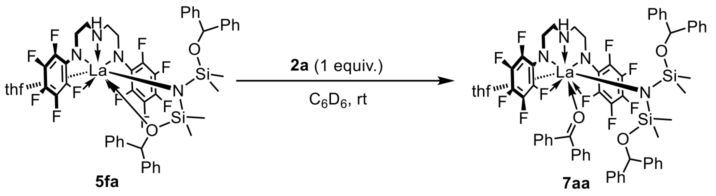

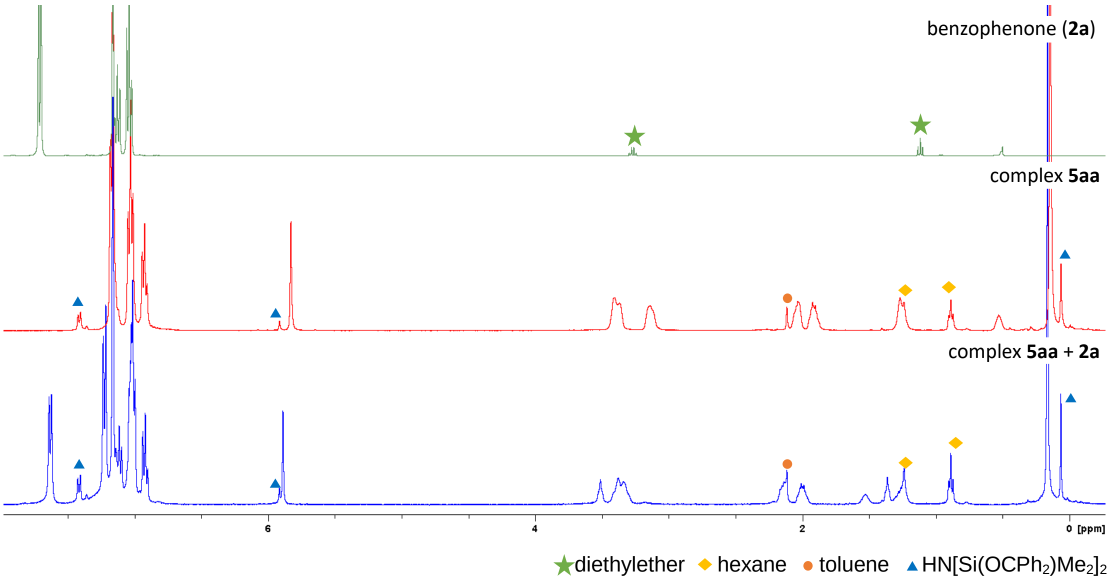

Figure S27. ^{1}H NMR spectra (400 MHz, C_6D_6, 30 °C) of benzophenone (**2a**) (*green*), complex **5aa** (*red*), and reaction mixture of **5aa** with 1 equiv of **2a** (*blue*).

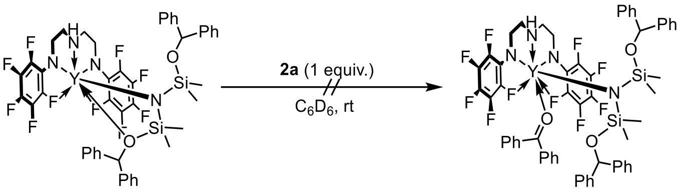

5fa **7fa**

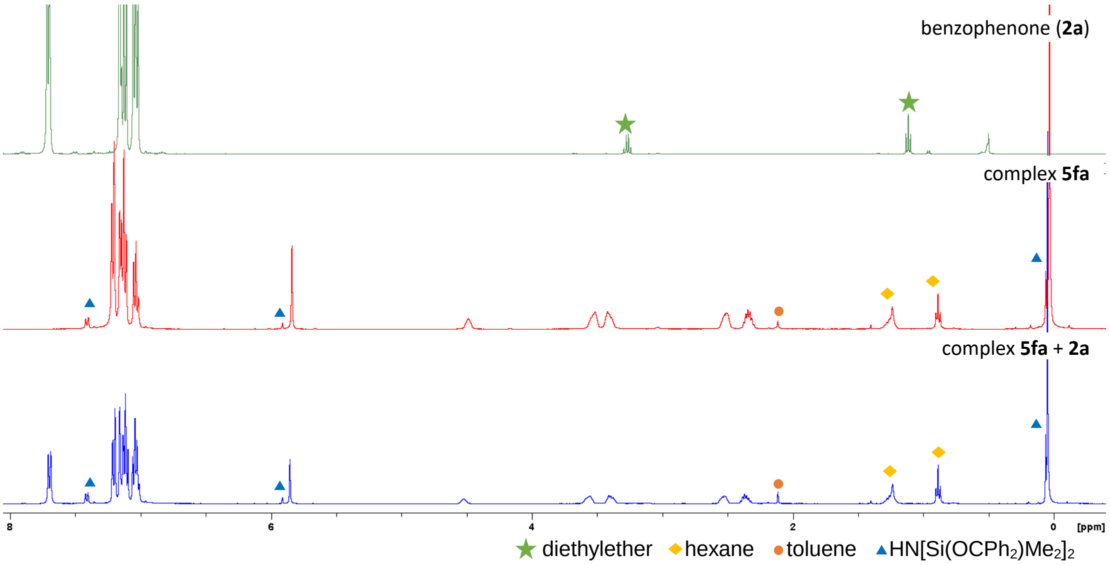

Figure S28. ^{1}H NMR spectra (400 MHz, C_6D_6, 30 °C) of benzophenone (**2a**) (*green*), complex **5fa** (*red*), and reaction mixture of **5fa** with 1 equiv of **2a** (*blue*).

XI. Kinetic Study

Normalized Time Scale Analysis for the Hydrosilylation Catalyzed by 4a

We determined a power value on the concentration of $HN(SiHMe_2)_2$ using time normalization analysis. In an Ar-filled glove box, to a solution of **4a** (5.0×10^{-3} mmol) in C_6D_6 (0.25 mL) placed in a vial was added a solution of **2a** (0.100 mmol) in C_6D_6 (0.25 mL) at ambient temperature. $HN(SiHMe_2)_2$ (17.3 µL) and hexamethylbenzene as an internal standard were added. The reaction mixture was transferred to a J-Young NMR tube. The yield of $HN[Si(OCHPh_2)Me_2]_2$ was determined by ^{1}H NMR measurement. The same operations were conducted for the reaction mixture containing $HN(SiHMe_2)_2$ (0.01 M and 0.015 M). As shown in Figure S29, the concentration of $HN[Si(OCHPh_2)Me_2]_2$ was plotted against a normalized time scale, t[**4a**]^a, and the power value, a, was adjusted until all the corrected yield curves overlay. We determined that the value of a was 1.

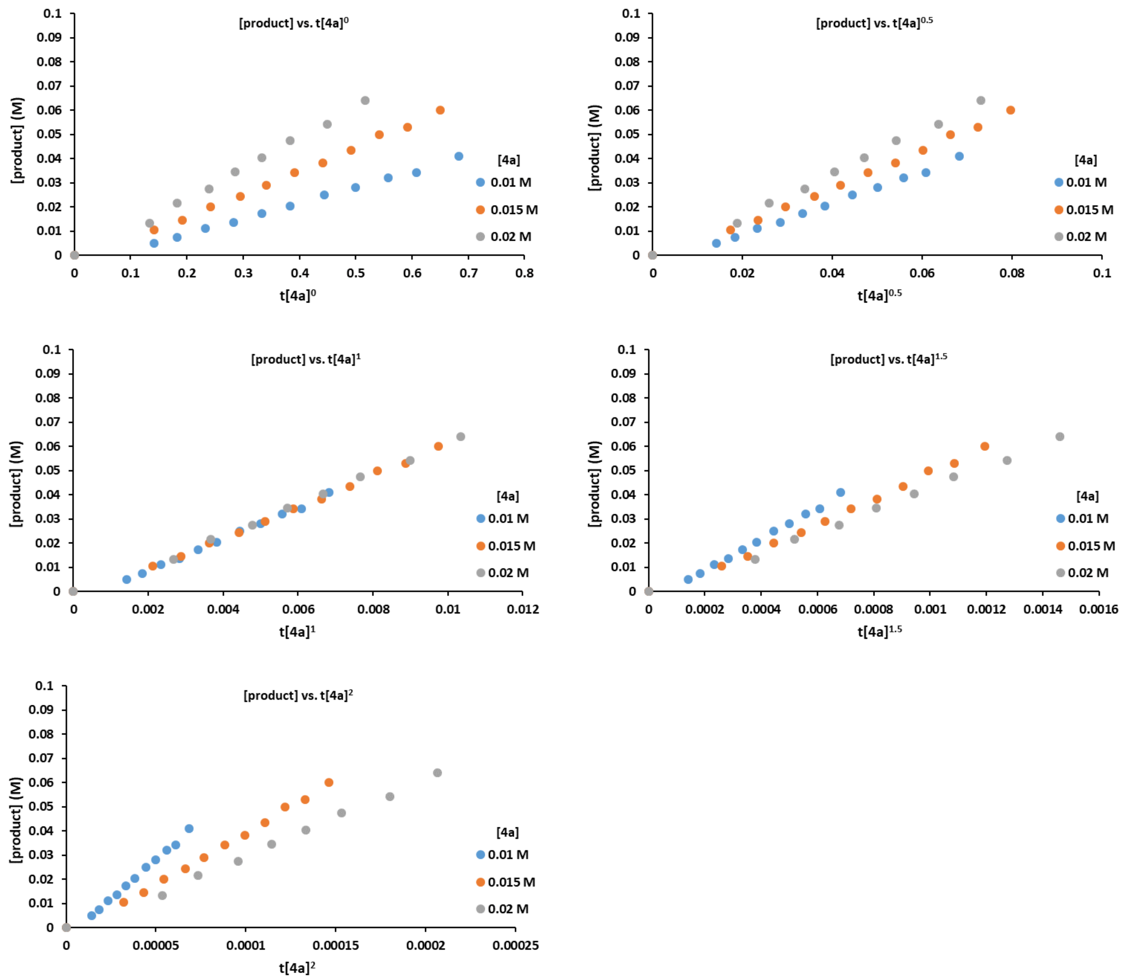

Figure S29. Normalized time scale analysis of the catalytic hydrosilylation of benzophenone to determine the order in catalyst **4a**.

Normalized Time Scale Analysis for $HN(SiHMe_2)_2$

We determined a power value on the concentration of $HN(SiHMe_2)_2$ using time normalization analysis. In an Ar-filled glove box, to a solution of **4a** (5.0×10^{-3} mmol) in C_6D_6 (0.25 mL) placed in a vial was added a solution of **2a** (0.100 mmol) in C_6D_6 (0.25 mL) at ambient temperature. $HN(SiHMe_2)_2$ (17.3 µL) and hexamethylbenzene as an internal standard were added. The reaction mixture was transferred to a J-Young NMR tube. The yield of $HN[Si(OCHPh_2)Me_2]_2$ was determined by ^1H NMR measurement. The same operations were conducted for the reaction mixture containing $HN(SiHMe_2)_2$ (0.15 M and 0.25 M). As shown in Figure S30, the concentration of $HN[Si(OCHPh_2)Me_2]_2$ was plotted against a normalized time scale, $\sum[HN(SiHMe_2)_2]^a\Delta t$, and the power value, *a*, was adjusted until all the corrected yield curves overlay. We determined that the value of *a* was 1.

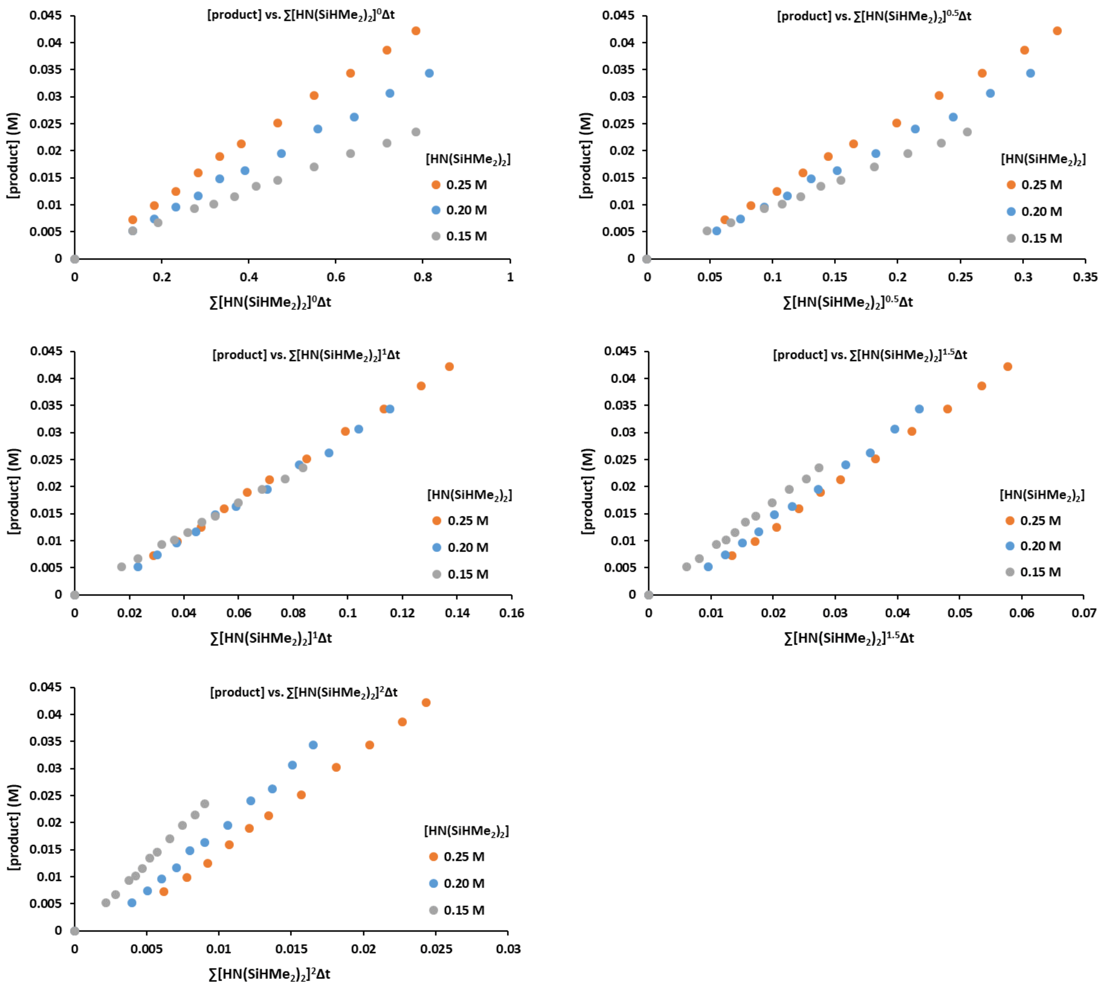

Figure S30. Normalized time scale analysis of the catalytic hydrosilylation of benzophenone to determine the order in $HN(SiHMe_2)_2$.

Normalized Time Scale Analysis for Benzophenone 2a

We determined a power value on the concentration of **2a** using time normalization analysis. In an Ar-filled glove box, to a solution of **4a** (5.0 x 10^{-3} mmol) in C_6D_6 (0.25 mL) placed in a vial was added a solution of **2a** (0.100 mmol) in C_6D_6 (0.25 mL) at ambient temperature. $HN(SiHMe_2)_2$ (17.3 μL) and hexamethylbenzene as an internal standard were added. The reaction mixture was transferred to a J-Young NMR tube. The yield of $HN[Si(OCHPh_2)Me_2]_2$ was determined by ^1H NMR measurement. The same operations were conducted for the reaction mixture containing **2a** (0.17 M and 0.23 M). As shown in Figure S31, the concentration of $HN[Si(OCHPh_2)Me_2]_2$ was plotted against a normalized time scale, $\sum[\mathbf{2a}]^a\Delta t$, and the power value, *a*, was adjusted until all the corrected yield curves overlay. We determined that the value of *a* was -2.

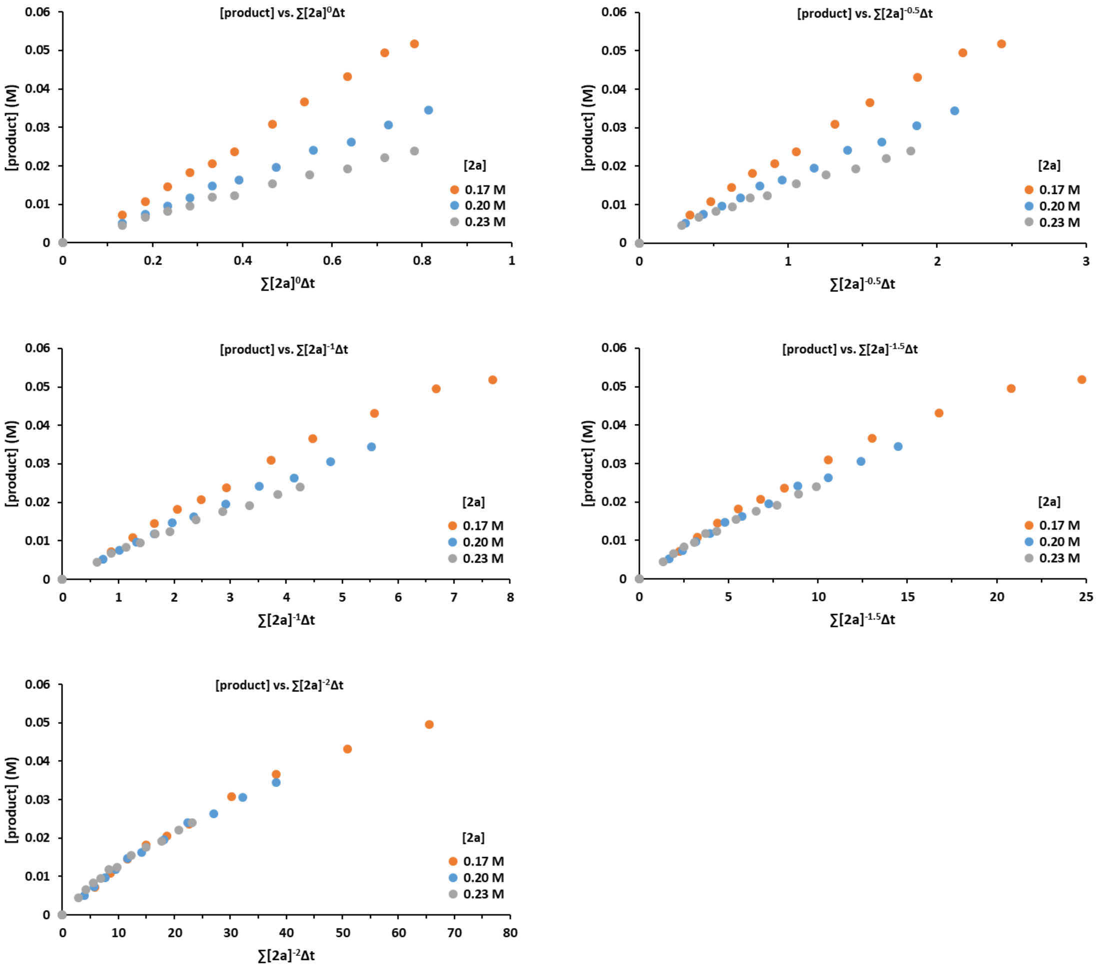

Figure S31. Normalized time scale analysis of the catalytic hydrosilylation of benzophenone to determine the order in **2a**.

XII. The Formation of 2k-Coordinated Adduct 8ak

To a solution of **4a** (6.43×10^{-3} mmol) in C_6D_6 (0.25 mL) was added a solution of **2k** (1.28×10^{-2} mmol) in C_6D_6 (0.25 mL) at ambient temperature. The color of reaction mixture immediately changed from colorless to dark orange. The ^1H NMR spectrum for reaction mixture is shown in Figure S32.

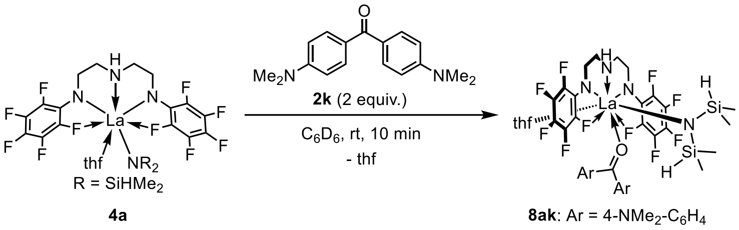

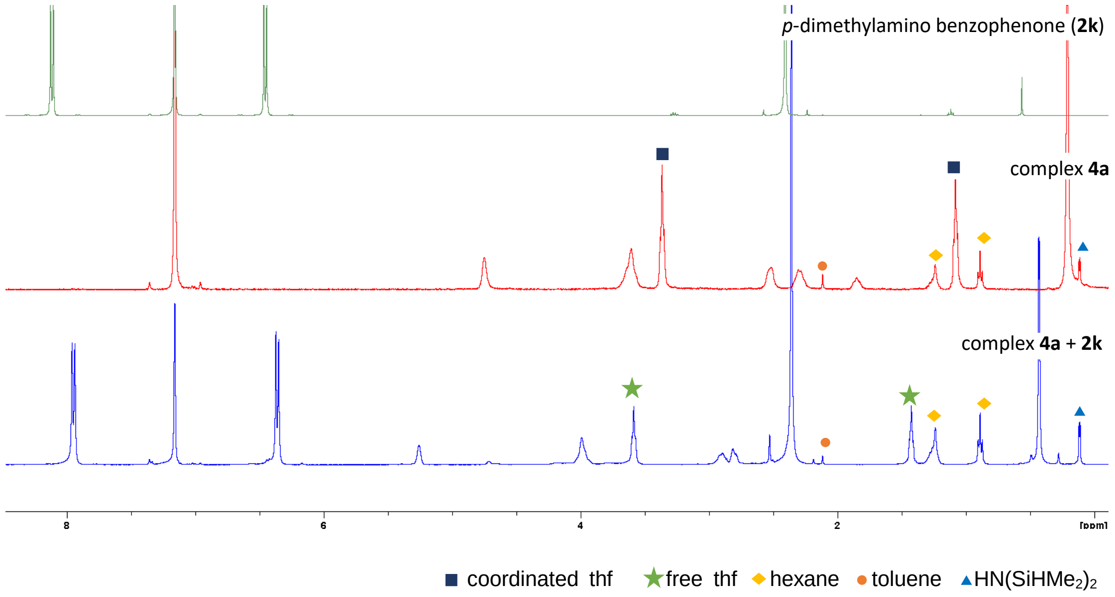

Figure S32. ^1H NMR spectra (400 MHz, C_6D_6, 30 °C) of *p*-dimethylamino benzophenone (**2k**) (*green*), complex **4a** (*red*) and reaction mixture of **4a** with 2 equiv of **2k** (*blue*).

XIII. Spectral Data for Diarymethanol Derivatives

^{1}H NMR (400 MHz, $CDCl_3$, 30 °C) for benzhydrol (**3a**)
δ 2.29 (brs, 1H, O*H*), 5.84 (s, 1H, OC*H*), 7.27—7.36 (m, 10H, *Ar*).

^{1}H NMR (400 MHz, $CDCl_3$, 30 °C) for bis(4-fluorophenyl)methanol (**3b**)
δ 2.31 (brs, 1H, O*H*), 5.81 (s, 1H, OC*H*), 7.02 (m, 4H, *Ar*), 7.32 (m, 4H, *Ar*).

^{1}H NMR (400 MHz, $CDCl_3$, 30 °C) for bis(4-chlorophenyl)methanol (**3c**)
δ 2.24 (brs, 1H, O*H*), 5.79 (s, 1H, OC*H*), 7.02 (m, 4H, *Ar*), 7.27 (m, 8H, *Ar*).

^{1}H NMR (400 MHz, $CDCl_3$, 30 °C) for bis(4-bromophenyl)methanol (**3d**)
δ 2.19 (brs, 1H, O*H*), 5.74 (s, 1H, OC*H*), 7.22 (d, 4H, *Ar*), 7.26 (d, 4H, *Ar*).

^{1}H NMR (400 MHz, $CDCl_3$, 30 °C) for bis(4-iodophenyl)methanol (**3e**)
δ 2.29 (brs, 1H, O*H*), 5.72 (s, 1H, OC*H*), 7.09 (d, 4H, *Ar*), 7.66 (d, 4H, *Ar*).

^{1}H NMR (400 MHz, $CDCl_3$, 30 °C) for bis(4-trifluorophenyl)methanol (**3f**)
δ 2.37 (brs, 1H, O*H*), 5.93 (s, 1H, OC*H*), 7.50 (d, 4H, *Ar*), 7.61 (d, 4H, *Ar*).

^{1}H NMR (400 MHz, $CDCl_3$, 30 °C) for bis(4-methylphenyl)methanol (**3g**)
δ 2.20 (brs, 1H, O*H*), 2.34 (s, 6H, *Me*), 5.79 (s, 1H, OC*H*), 7.15 (d, 4H, *Ar*), 7.27 (m, 4H, *Ar*).

^{1}H NMR (400 MHz, $CDCl_3$, 30 °C) for bis(4-*tert*-butylphenyl)methanol (**3h**)
δ 1.32 (s, 18H, ^{t}Bu) 2.24 (brs, 1H, O*H*), 5.81 (s, 1H, OC*H*), 7.35 (m, 8H, *Ar*).

^{1}H NMR (400 MHz, $CDCl_3$, 30 °C) for bis(4-methoxyphenyl)methanol (**3i**)
δ 2.58 (brs, 1H, O*H*), 5.75 (s, 1H, OC*H*), 6.86 (d, 4H, *Ar*), 7.27 (d, 4H, *Ar*).

XIV. ^{1}H NMR Spectra of Catalytic Reaction Mixtures

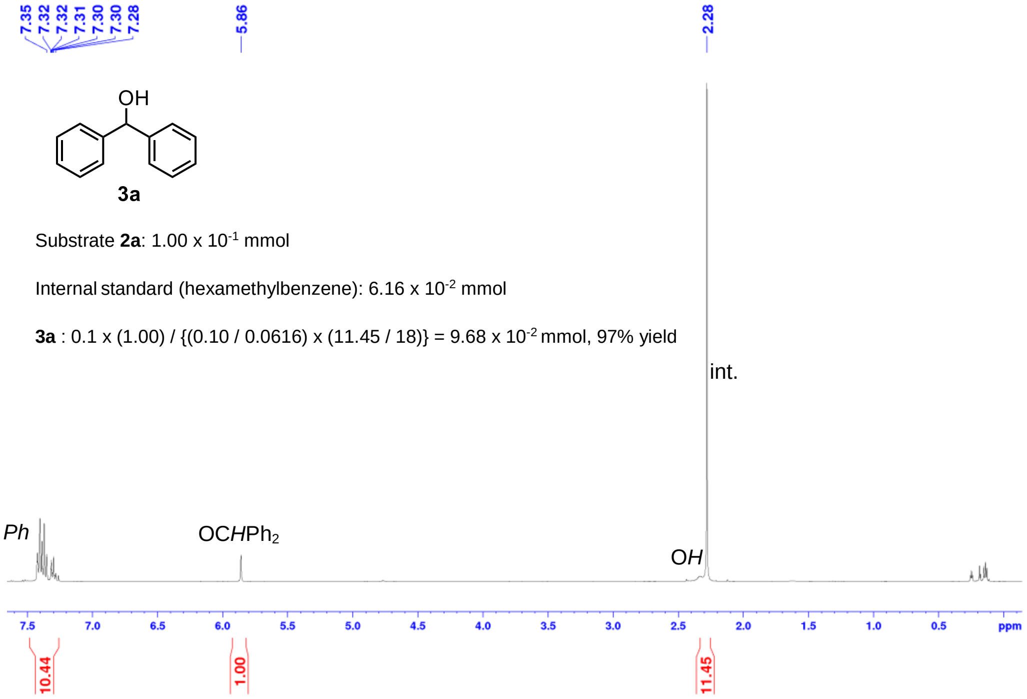

Figure S33. ^{1}H NMR spectrum (400 MHz, $CDCl_3$, 30 °C) of the reaction mixture for hydrosilylation of **2a**.

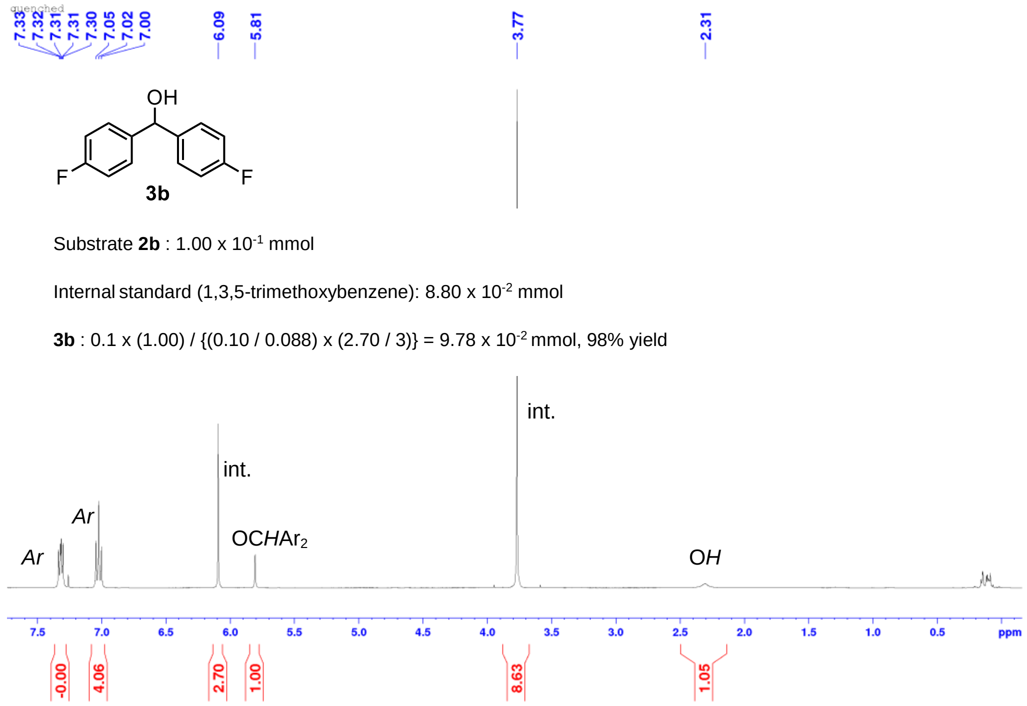

Figure S34. ^{1}H NMR spectrum (400 MHz, $CDCl_3$, 30 °C) of the reaction mixture for hydrosilylation of **2b**.

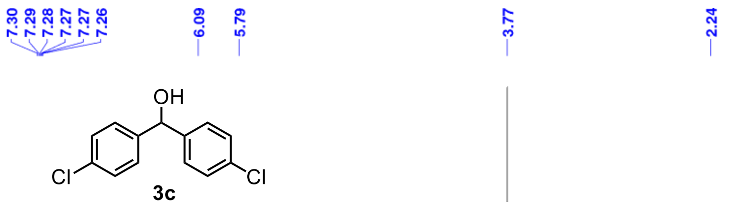

Substrate **2c** : 1.00 x 10^{-1} mmol

Internal standard (1,3,5-trimethoxybenzene): 6.30 x 10^{-2} mmol

3c : 0.1 x (1.00) / {(0.10 / 0.063) x (1.92 / 3)} = 9.84 x 10^{-2} mmol, 98% yield

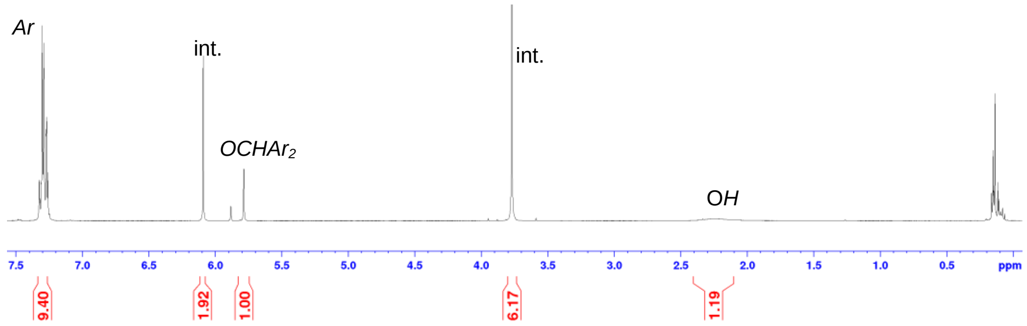

Figure S35. ^{1}H NMR spectrum (400 MHz, $CDCl_3$, 30 °C) of the reaction mixture for hydrosilylation of **2c**.

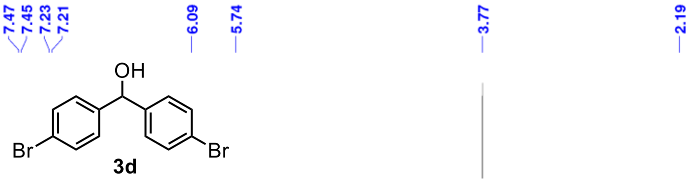

Substrate **2d** : 1.00 x 10^{-1} mmol

Internal standard (1,3,5-trimethoxybenzene): 8.50 x 10^{-2} mmol

3d : 0.1 x (1.00) / {(0.10 / 0.0850) x (2.73 / 3)} = 9.34 x 10^{-2} mmol, 93% yield

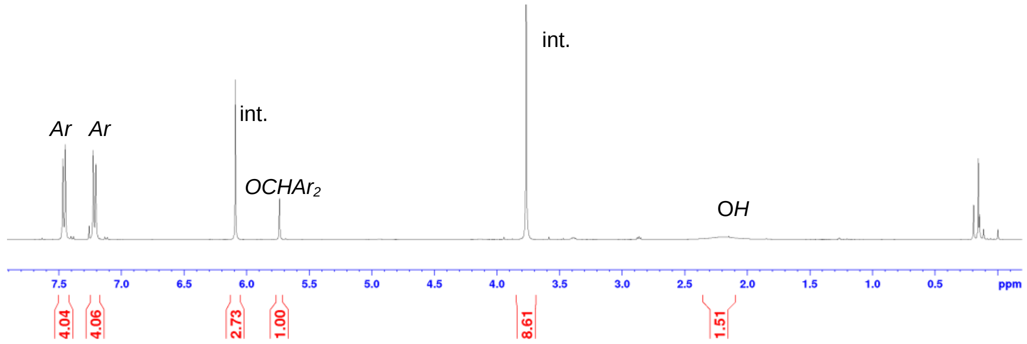

Figure S36. ^{1}H NMR spectrum (400 MHz, $CDCl_3$, 30 °C) of the reaction mixture for hydrosilylation of **2d**.

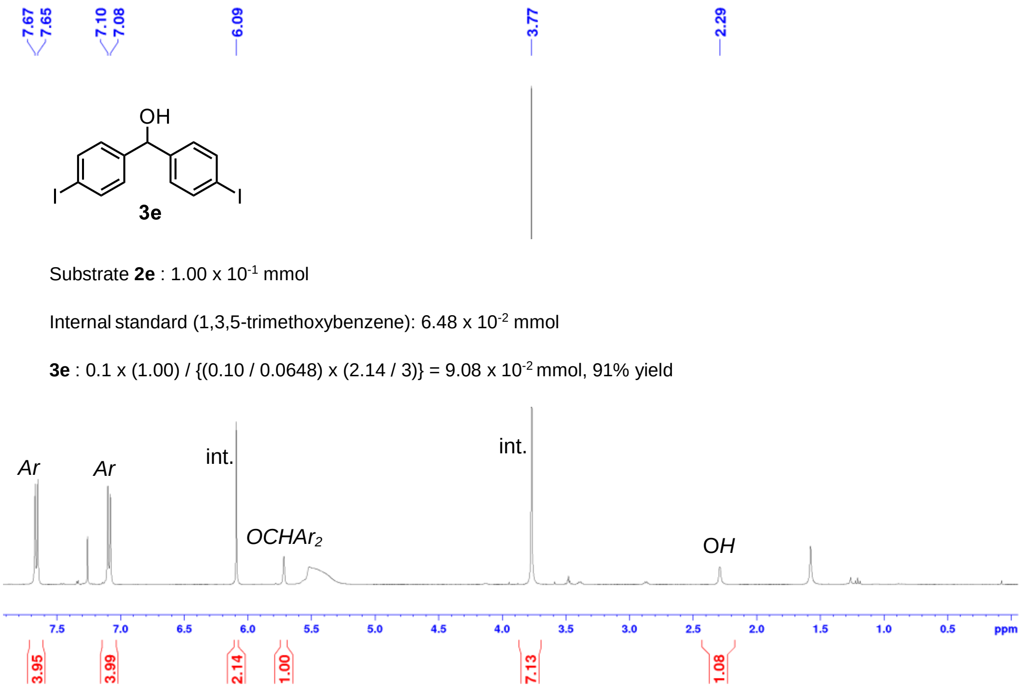

Figure S37. ^{1}H NMR spectrum (400 MHz, $CDCl_3$, 30 °C) of the reaction mixture for hydrosilylation of **2e**.

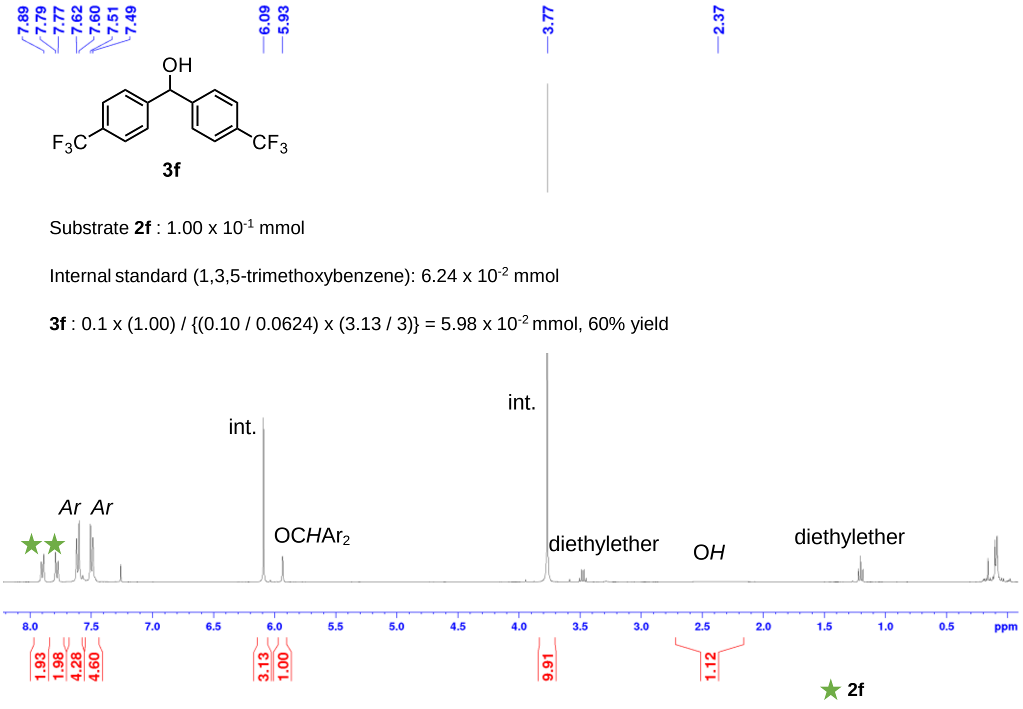

Figure S38. ^{1}H NMR spectrum (400 MHz, $CDCl_3$, 30 °C) of the reaction mixture for hydrosilylation of **2f**.

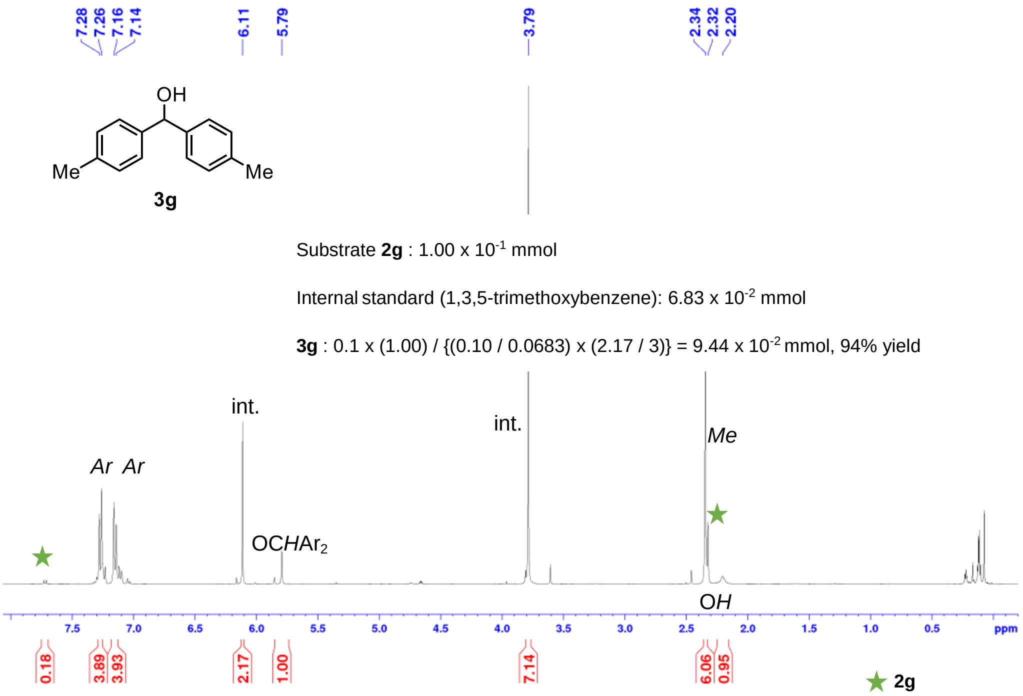

Figure S39. ^{1}H NMR spectrum (400 MHz, $CDCl_3$, 30 °C) of the reaction mixture for hydrosilylation of **2g**.

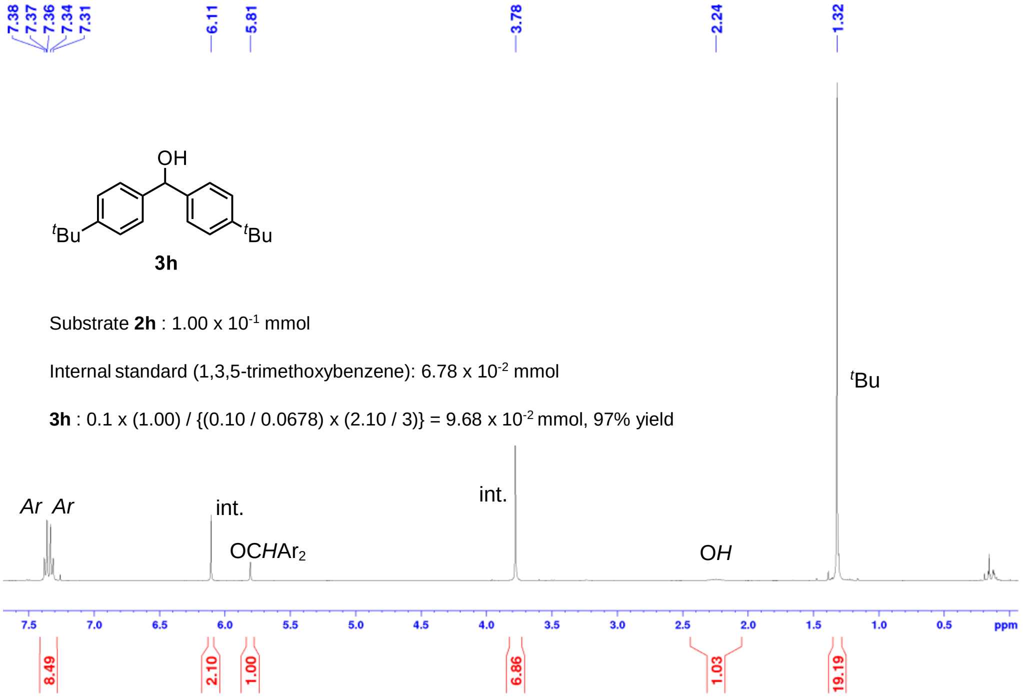

Figure S40. ^{1}H NMR spectrum (400 MHz, $CDCl_3$, 30 °C) of the reaction mixture for hydrosilylation of **2h**.

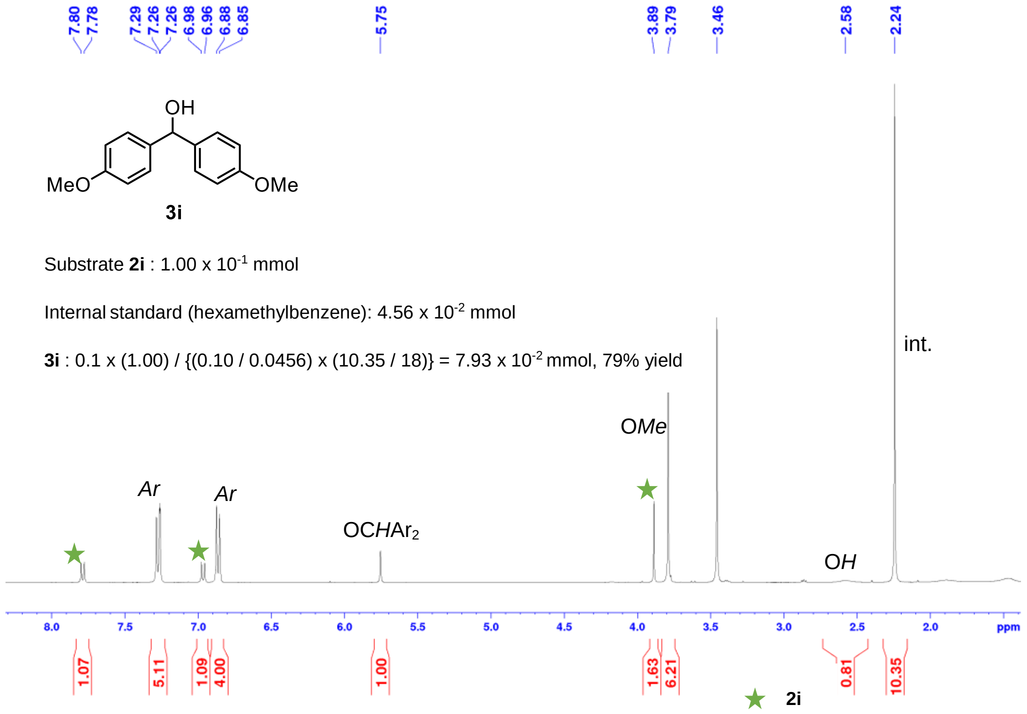

Figure S41. ^{1}H NMR spectrum (400 MHz, $CDCl_3$, 30 °C) of the reaction mixture for hydrosilylation of **2i**.

XV. Crystal Data and Data Collection Parameters

XV-1. Lanthanide Silylamide Complexes (**4a**, **4b,** and **4f**)

complex	**4a**	**4b**	**4f**
empirical fomula	$C_{24}H_{22}F_{10}LaN_4OSi_2$	$C_{24}H_{31}F_{10}CeN_4OSi_2$	$C_{27}H_{27}F_{10}YN_4OSi_2$
formula weight	767.52	777.81	758.60
cryst. system	Triclinic	Triclinic	Triclinic
space group	$P\bar{1}$ (#2)	$P\bar{1}$ (#2)	$P\bar{1}$ (#2)
a, Å	8.273(2)	8.2581(15)	9.7522(6)
b, Å	10.777(3)	10.828(2)	10.1776(7)
c, Å	18.574(5)	18.528(3)	18.6721(13)
a, deg	92.786(4)	92.687(3)	83.572(4)
b, deg	94.631(4)	94.979(4)	82.218(4)
g, deg	108.526(6)	109.109(5)	81.251(4)
V, Å^3	1560.2(7)	1554.6(5)	1806.9(2)
Z	2	2	2
Dcald, g/cm^3	1.634	1.662	1.394
μ [Mo-*K*α], cm^{-1}	15.246	16.258	17.599
T, K	113	113	113
crystal size	0.110 x 0.100 x 0.010	0.130 x 0.100 x 0.040	0.240 x 0.240 x 0.040
θ range for data collection (deg)	3.40, 26.60	3.30, 27.50	3.10, 27.50
no. of reflections measured	29026	37145	33590
Unique data (*R*int)	0.1142	0.0474	0.0740
data / restraints / parameters	7177/0/375	7142/0/397	8306/0/388
$R1$ ($I > 2.0\sigma(I)$) ^{a}	0.0647	0.0312	0.0410
$wR2$ ($I > 2.0\sigma(I)$) ^{b}	0.1433	0.0778	0.1246
R1 (all data) ^{a}	0.0986	0.0397	0.0550
$wR2$ (all data) ^{b}	0.1582	0.0801	0.1439
GOF on F^2	1.099	1.037	0.586
Δρ, e Å^{-3}	1.96, -0.78	1.31, -0.69	0.69, -0.82

(*a*) $R1 = (\Sigma||Fo| - |Fc||)/(\Sigma|Fo|)$, (*b*) $wR2 = [\{\Sigma w(Fo^2 - Fc^2)^2\}/\{\Sigma w(Fo^4)\}]^{1/2}$

XV-2. Lanthanide Silylamide Complexes (**5aa** and **5ba**)

complex	**5aa**	**5ba**
empirical fomula	$C_{63}H_{64}F_{10}LaN_4O_3Si_2$	$C_{64}H_{67}CeF_{10}N_4O_3Si_2$
formula weight	1310.28	1326.53
cryst. system	Monoclinic	Monoclinic
space group	$P2_1/c$ (#14)	$P2_1/c$ (#14)
a, Å	17.635(3)	17.614(3)
b, Å	18.761(3)	18.687(3)
c, Å	19.853(3)	19.833(4)
a, deg	-	-
b, deg	108.133(3)	107.973(3)
g, deg	-	-
V, $Å^3$	6242.2(14)	6209.5(19)
Z	4	4
Dcald, g/cm^3	1.394	1.419
μ [Mo-*K*α], cm^{-1}	7.973	8.493
T, K	113	113
crystal size	0.360 x 0.260 x 0.030	0.130 x 0.130 x 0.030
θ range for data collection (deg)	3.00, 27.40	3.10, 27.50
no. of reflections measured	141379	139279
Unique data (*R*int)	0.2028	0.1732
data / restraints / parameters	14485/0/748	14321/0/757
$R1$ ($I > 2.0\sigma(I)$) [a]	0.0725	0.0669
$wR2$ ($I > 2.0\sigma(I)$) [b]	0.2045	0.1372
R1 (all data) [a]	0.1132	0.1062
$wR2$ (all data) [b]	0.1132	0.1568
GOF on F^2	0.987	1.110
Δρ, e $Å^{-3}$	3.04, -2.88	2.21, -1.32

(*a*) $R1 = (\Sigma||Fo| - |Fc||)/(\Sigma|Fo|)$, (*b*) $wR2 = [\{\Sigma w(Fo^2 - Fc^2)^2\}/\{\Sigma w(Fo^4)\}]^{1/2}$

XVI. Molecular Structure of 4b and 5ba

Ce(**L**^{1})[N($SiHMe_2$)$_2$](thf) (**4b**)

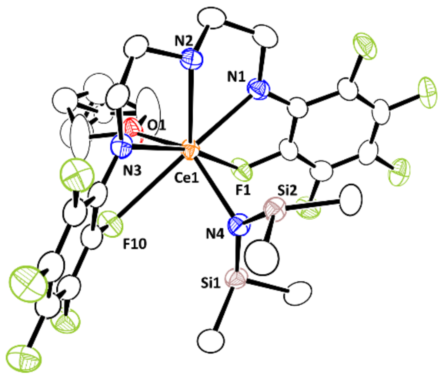

Figure S42. ORTEP drawing of the crystal structure of **4b** with ellipsoids shown at 50% probability. All hydrogen atoms and solvent molecules are omitted for clarity. Selected interatomic distances (Å): Ce1—N1, 2.455(2); Ce1—N2, 2.586(2); Ce1—N3, 2.467(2); Ce1—N4, 2.335(2); Ce1—F10, 2.772(2); Ce1—F1, 2.739(2); Ce1—O1, 2.511(2); Ce1—Si1, 3.517(2); Ce1—Si2, 3.229(1). Selected interatomic angles (°): Ce1—N4—Si1, 120.8(1); Ce1—N4—Si2, 105.4(1).

Ce(**L**^{1})[N[Si(OCHPh$_2$)Me$_2$]$_2$](thf) (**5ba**)

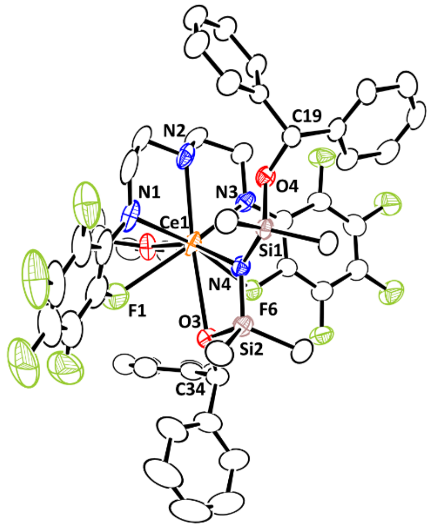

Figure S43. ORTEP drawing of the crystal structure of **5ba** with ellipsoids shown at 50% probability. All hydrogen atoms and solvent molecules are omitted for clarity. Selected interatomic distances (Å): Ce1—N1, 2.485(5); Ce1—N2, 2.581(5); Ce1—N3, 2.511(5); Ce1—N4, 2.368(4); Ce1—F1, 2.766(4); Ce1—F6, 2.675(3); Ce1—O3, 2.847(4); Ce1—O4, 3.527(3); C34—O3, 1.454(6); C19—O4, 1.441(6). Selected interatomic angles (°): Ce1—N4—Si1, 123.7(2); Ce1—N4—Si2, 109.4(2).

[References]
[S1] A. B. Pangborn, M. A. Giardello, R. H. Grubbs, R. K. Rosen and F. J. Timmers, Safe and Convenient Procedure for Solvent Purification. *Organometallics*, 1996, **15**, 1518—1520.
[S2] R. Anwander, O. Runte, J. Eppinger, G. Gerstberger, E. Herdtweck and M. Spiegler, *J. Chem. Soc., Dalton Trans.*, 1998, 847—858.
[S3] R. R. Schrock, J. Lee, L.-C. Liang and W. M. Davis, *Inorg. Chim. Acta.*, 1998, **270**, 353—362.
[S4] F. V. Cochran, P. J. Bonitatebus and R. R. Schrock, *Organometallics*, 2000, **13**, 2414—2416.
[S5] R. Cowdell, C. J. Davies, S. J. Hilton, J.-D. Maréchal, G. A. Solan, O. Thomas and J. Fawcett, *Dalton Trans.*, 2004, 3231—3240.
[S6] C. J. Davies, S. J. Hilton, G. A. Solan, W. Stannard and J. Fawcett, *Polyheedron*, 2005, **24**, 2017 2016.
[S7] A. G. Avent, F. G. N. Cloke, B. R. Elvidge and P. B. Hitchcock, *Dalton Trans.*, 2004, 1083—1096.
[S8] G. A. Grasa, M. S. Viciu, J. Huang and S. P. Nolan, *J. Org. Chem.*, 2001, **66**, 7729—7737.
[S9] E. Blom, F. Karimi and B. Långström, *J. Label Compd. Radiopharm*, 2009, **52**, 504—511.
[S10] K. Albrecht and K. Yamamoto, *J. Am. Chem. Soc.*, 2009, **131**, 2244—2251.
[S11] H. K. Ulbrich, A. Luxenburger, P. Prech, E. E. Eriksson, O. Soehnlein, P. Rotzius, L. Lindbom and G. Dannhardt, *J. Med. Chem.*, 2006, **49**, 5988—5999.
[S12] Y. Shen, Y. Gu and R. Martin, *J. Am. Chem. Soc.*, 2018, **140**, 12200—12209.
[S13] van der Sluis, P.; Spek, A.L. BYPASS: an Effective Method for the Refinement of Crystal Structures Containing Disordered Solvent Regions. Acta Cryst. 1990, A46, 194—201.
